# High levels of *NRF2* sensitize temozolomide-resistant glioblastoma cells to ferroptosis via ABCC1/MRP1 upregulation

**DOI:** 10.1038/s41419-022-05044-9

**Published:** 2022-07-08

**Authors:** I. de Souza, L. K. S. Monteiro, C. B. Guedes, M. M. Silva, M. Andrade-Tomaz, B. Contieri, M. T. Latancia, D. Mendes, B. F. M. M. Porchia, M. Lazarini, L. R. Gomes, C. R. R. Rocha

**Affiliations:** 1 Department of Clinical and Experimental Oncology, Federal University of São Paulo (UNIFESP), São Paulo, 04037-003 Brazil; 2 Institute of Biomedical Science, University of São Paulo (USP), São Paulo, 04037-003 Brazil; 3 Department of Pharmaceutical Sciences, Federal University of São Paulo (UNIFESP), São Paulo, 04037-003 Brazil; 4 Cell Cycle Laboratory, Butantan Institute, São Paulo, Brazil

**Keywords:** Cancer genetics, Cell death

## Abstract

Glioblastoma patients have a poor prognosis mainly due to temozolomide (TMZ) resistance. *NRF2* is an important transcript factor involved in chemotherapy resistance due to its protective role in the transcription of genes involved in cellular detoxification and prevention of cell death processes, such as ferroptosis. However, the relation between *NRF2* and iron-dependent cell death in glioma is still poorly understood. Therefore, in this study, we analyzed the role of *NRF2* in ferroptosis modulation in glioblastoma cells. Two human glioblastoma cell lines (U251MG and T98G) were examined after treatment with TMZ, ferroptosis inducers (Erastin, RSL3), and ferroptosis inhibitor (Ferrostatin-1). Our results demonstrated that T98G was more resistant to chemotherapy compared to U251MG and showed elevated levels of *NRF2* expression. Interestingly, T98G revealed higher sensitivity to ferroptosis, and significant GSH depletion upon system xc^−^ blockage. *NRF2* silencing in T98G cells (T98G-sh*NRF2*) significantly reduced the viability upon TMZ treatment. On the other hand, T98G-sh*NRF2* was resistant to ferroptosis and reverted intracellular GSH levels, indicating that *NRF2* plays a key role in ferroptosis induction through GSH modulation. Moreover, silencing of *ABCC1*, a well-known *NRF2* target that diminishes GSH levels, has demonstrated a similar collateral sensitivity. T98G-si*ABCC1* cells were more sensitive to TMZ and resistant to Erastin. Furthermore, we found that *NRF2* positively correlates with *ABCC1* expression in tumor tissues of glioma patients, which can be associated with tumor aggressiveness, drug resistance, and poor overall survival. Altogether, our data indicate that high levels of *NRF2* result in collateral sensitivity on glioblastoma via the expression of its pro-ferroptotic target *ABCC1*, which contributes to GSH depletion when the system xc^−^ is blocked by Erastin. Thus, ferroptosis induction could be an important therapeutic strategy to reverse drug resistance in gliomas with high *NRF2* and *ABCC1* expression.

## Introduction

Gliomas are the most common cancers of the central nervous system (CNS), representing approximately 50% of all brain tumors [1]. Glioblastoma (GBM, WHO grade IV glioma) is the most aggressive type of glioma, and it is the main lethal type of primary malignant tumor in adults [2, 3]. The standard therapy—which includes surgery, radiotherapy, and/or concomitant adjuvant chemotherapy—has low efficacy due to tumor heterogeneity [1]. Consequently, the overall survival rate of glioblastoma patients is only 12–15 months [2]. Albeit improving survival for about 2.5 months, chemotherapy has limited success due to drug resistance to the main agent used in clinical practice, temozolomide (TMZ) [2, 4, 5]. Thus, reversing this resistance is a critical challenge in medical sciences.

Previous studies have shown TMZ resistance as a multifactorial process that occurs by a variety of cell mechanisms, including the increase in the transcription factor *NRF2* (nuclear factor erythroid 2-related factor 2) expression [6, 7]. *NRF2* is involved in chemotherapy resistance through the regulation of several antioxidant genes [8]. Indeed, it is known that *NRF2* upregulation promotes resistance to cisplatin [9], doxorubicin, etoposide [10], and TMZ in cancer cells [11]. Considering that protection against cell death events has an important role in drug resistance, induction of regulated cell death is a potential target for cancer therapy. In that sense, ferroptosis induction has been widely studied as a potential therapeutic target in glioblastoma treatment to reverse chemoresistance and predict prognosis [12–14].

Ferroptosis is a nonapoptotic, iron-dependent form of cell death characterized by the loss of lipid peroxide repair capacity by the glutathione peroxidase 4 (GPX4); polyunsaturated fatty acid oxidation, and availability of redox-active iron [15, 16]. Notwithstanding, there is a lack of knowledge regarding the role of ferroptosis in brain tumors. Notably, *NRF2* is a regulator of a range of antioxidant genes, such as the xc- cystine-glutamate antiporter (*SLC7A11*), which promotes cystine uptake for glutathione (GSH) synthesis [17, 18], and GPX4, which uses GSH as a cofactor to reduce lipid peroxides to lipid alcohols preventing ferroptosis [19, 20]. Therefore *NRF2* expression has been related to the modulation of ferroptosis [21]. Indeed, *NRF2* was considered a negative regulator of ferroptosis since inhibition of this gene sensitizes head and neck cancer cells to ferroptosis and its high expression promotes resistance in glioma cells [21–23].

On the other hand, recent studies in lung cancer cells have shown that *NRF2* upregulation sensitizes tumor cells to ferroptosis through the increase of MRP1 expression [24]. Similarly, high *NRF2* activity promoted high levels of heme oxygenase 1 (*HMOX1*), which acts detoxifying heme into biliverdin, releasing redox-active iron and, consequently, inducing lipid peroxidation in neuroblastoma [25] and fibrosarcoma cells [26]. Hence, the modulation of ferroptosis on glioblastoma still needs to be elucidated.

With that in mind, we have investigated these contrasting roles of *NRF2* in ferroptosis in glioblastoma cell lines to identify new therapeutical strategies—via ferroptosis induction—to sensitize chemoresistant cell lines and induce tumor cell death. In addition, we have analyzed the gene expression of glioma patients from GlioVis database to support our findings. Altogether, our data suggest that high expression of *NRF2* may result in ferroptosis sensitivity on TMZ-resistant glioblastoma through elevated levels of expression of its pro-ferroptotic target *ABCC1*, which contributes to GSH depletion when system xc^−^ is blocked. Also, TMZ-sensitive cells can present ferroptosis resistance through GSH modulation.

## Results

### T98G cell line is more resistant to TMZ treatment in comparison to U251MG

**Fig. 1:**
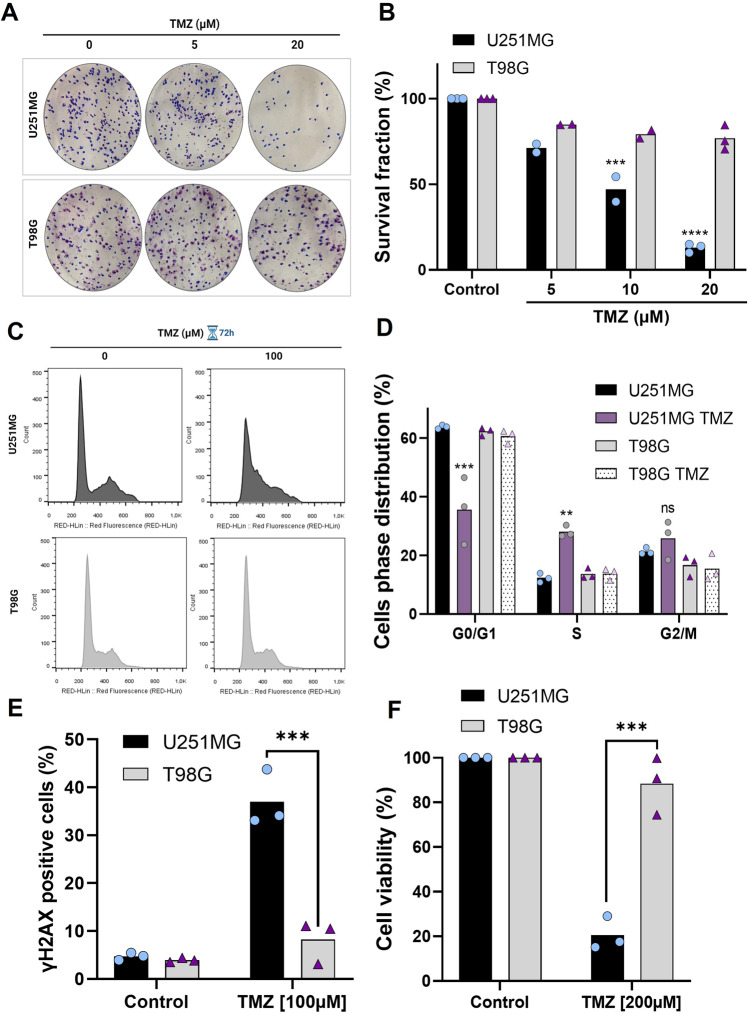
Differential response of glioma cells to TMZ treatment. **A** Representative images of colonies formed in each group treated with 5 and 20 µM TMZ. **B** Quantification of total colonies after TMZ treatment (5, 10, and 20 µM) in comparison to controls. **C** Histograms represent cell cycle distribution with TMZ treatment for 72 h. **D** Cell cycle distribution was conducted by flow cytometer analysis after TMZ treatment (100 μM) for 72 h. **E** Flow cytometry analysis of the percentage of γH2AX positive cells upon treatment with TMZ (100 μM) for 48 h. **F** Cell viability was analyzed 120 h after treatment with TMZ (200 μM) and measured by XTT assay. Values are mean ± SEM of three independent experiments, ns = not statistically significant, *P < 0.05, **P < 0.01, ***P < 0.001, ****P < 0.0001. Each dot represents an independent experiment.

Glioblastoma cell lines may be resistant to TMZ-induced cell death by multiple molecular mechanisms [6, 27]. Using the human glioblastoma cell lines U251MG and T98G we first confirmed their sensitivity pattern to TMZ, initially performing a clonogenic assay. U251MG cells displayed a greater sensitivity to TMZ treatment when compared to T98G cells (Fig. 1A, B). Cell cycle analysis, using flow cytometry, showed that treatment with TMZ resulted in cell cycle arrest in the S phase in U251MG, but not in T98G cells (Fig. 1C, D). We also observed in that TMZ treatment resulted in a higher amount of DNA damage in U251MG cells when compared to T98G cells (Fig. 1E). Similarly, U251MG showed significant viability reduction after TMZ treatment compared to T98G, which was more resistant to the chemotherapeutic drug with 80% viable cells, as shown by XTT cell viability assay (Fig. 1F). These data confirmed the accentuated resistance to TMZ treatment of T98G cells.

### T98G cells have higher *NRF2* expression

**Fig. 2:**
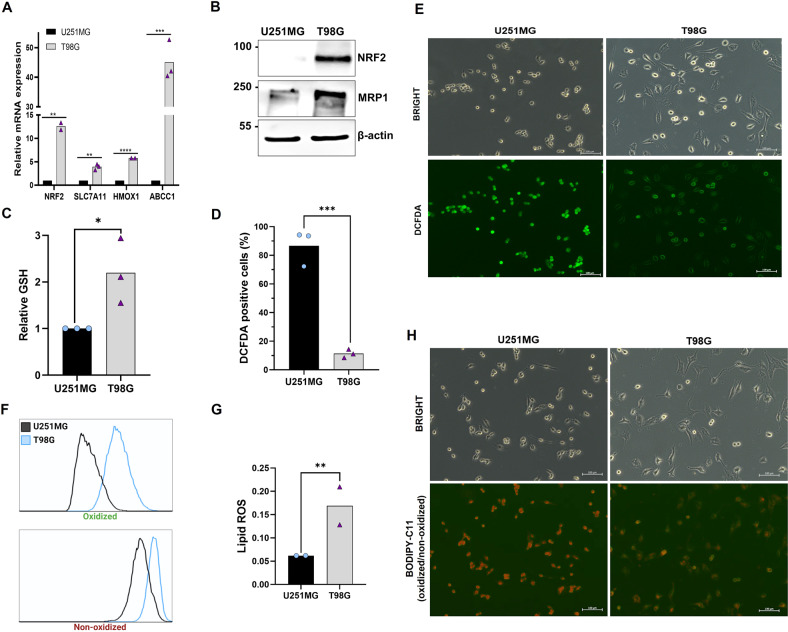
NRF2 and lipoperoxidation levels in glioblastoma. **A** Quantitative real-time PCR analysis of NRF2, SLC7A11, HMOX1, and *ABCC1* mRNA levels in glioblastoma cells. **B** Detection of NRF2 and MRP1 protein by western blot in U251MG and T98G cell lines. **C** Quantification of basal intracellular GSH in both glioblastoma cells. **D** Basal level of ROS detected by DCFDA probe and analyzed by flow cytometry. **E** Fluorescence microscopy representative images of basal DCFDA fluorescence in U251MG and T98G. Scale bar: 100 μm. **F** Representative histograms of lipid peroxidation in U251MG and T98G. Cellular stress was induced by RSL3 (1 μM) treatment and lipid peroxidation was measured by BODIPY-C11 581/591 probe by flow cytometry (**G**) and the ratio of oxidized/non-oxidized cells was calculated. **H** Fluorescence microscopy representative images of basal BODIPY-C11 fluorescence in U251MG and T98G. Scale bar: 100 μm. Values are mean ± SEM of two or three independent experiments, ns = not statistically significant, *P < 0.05, **P < 0.01, ***P < 0.001, ****P < 0.0001. Each dot represents an independent experiment.

*NRF2* upregulation promotes drug resistance in several cancer cells lines [9, 28, 29], including glioma cells [11], through the modulation of intracellular redox homeostasis and drug detoxification [30]. Indeed, T98G cells displays of *NFE2L2* (gene that encodes NRF2) amplification, and therefore it presents high levels of NRF2 [31]. In order to investigate whether *NRF2* expression could be involved with T98G TMZ-resistance, we performed quantitative real-time PCR analysis to measure *NRF2* expression and its target genes in both cell lines. First, as expected, we observed a much higher NRF2 mRNA expression in T98G cells when compared to U251MG (about 12-fold increase) (Fig. 2A). Likewise, the TMZ-resistant cell line showed higher mRNA levels of *NRF2* targets genes, such as the solute carrier family 7 member 11 (*SLC7A11*), heme oxygenase 1 (*HMOX1*), and ATP binding cassete subfamily C member 1 (*ABCC1*) (Fig. 2A). High levels of NRF2 and MRP1 protein expression in T98G cells were also confirmed by western blot analysis (Fig. 2B).

Since *NFR2* is directly related to GSH synthesis, we quantified GSH levels in both cell lines, and T98G cells showed increased levels of GSH compared to U251MG (Fig. 2C). And once GSH is the major molecule of antioxidant system, and plays a crucial role in the prevention of reactive oxygen species (ROS) accumulation [32], we quantified the total levels of ROS by DCFDA fluorescence in both cell lines. Using flow cytometry analysis, we observed that T98G cells present 8-fold less ROS than U251MG cells (Fig. 2D). In accordance, fluorescence microcopy indicated lower levels of ROS in T98G cells, when compared to U251MG (Fig. 2E).

Considering that the role of *NRF2* in ferroptosis remains largely unclear, using two glioma cell lines with different expressions of *NRF2* could be a great strategy to explore the relation between *NRF2* and iron-dependent cell death. Thus, to verify ferroptosis induction in these cells we initially analyzed the differential lipoperoxidation levels in both cell lines after stress induced by RSL3, a well-known ferroptosis inducer. We observed, by flow cytometry and fluorescence microscopy with BODIPY-C11 581/591 probe that the TMZ-resistant cell line T98G generates more lipid ROS when compared to U251MG cells (Fig. 2F–H).

### TMZ-resistant cell line is sensitive to ferroptosis

**Fig. 3:**
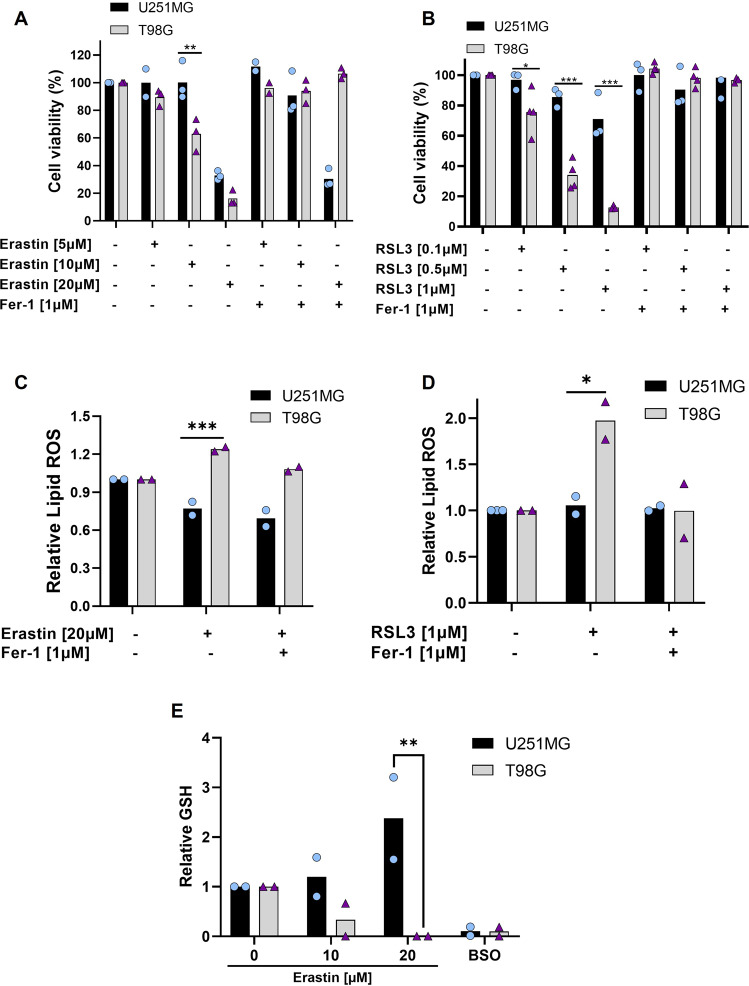
Differential sensitivity of glioma cells to ferroptosis. **A** Cells were treated with Erastin (5, 10, and 20 μM) and Ferrostatin-1 (1 μM) for 72 h, and cell viability was measured by XTT assay. **B** Cell viability under another ferroptosis inducer, RSL3 (0.1, 0.5, and 1 μM), and Ferrostatin-1 (1 μM) for 72 h was tested by XTT assay. **C** Cells were treated with Erastin (20 μM) and Ferrostatin-1 (1 μM), after 24 h lipid ROS was detected by BODIPY-C11 probe (ratio oxidized/non-oxidized) and analyzed by flow cytometry. **D** Lipid ROS also was measured by the BODIPY-C11 probe (ratio oxidized/non-oxidized) after RSL3 (1 μM) and Fer-1 (1 μM) treatment for 24 h. **E** Intracellular GSH level quantification after treatment with Erastin (10 and 20 μM) and BSO (1 mM) for 24 h. Values are mean ± SEM of two or three independent experiments, ns = not statistically significant, *P < 0.05, **P < 0.01, ***P < 0.001, ****P < 0.0001. Each dot represents an independent experiment.

These observations led us to investigate whether ferroptosis could be induced both in TMZ-sensitive and resistant glioblastoma cell lines. Thus, the cells were treated with two ferroptosis inducers: Erastin (a system xc^-^ inhibitor blocking cystine uptake), and RSL3 (a GPX4 inhibitor) [15]. By analyzing cell viability, we surprisingly found that T98G cells were extremely responsive to Erastin and RSL3 in all administered doses, while U251MG was more resistant to ferroptosis (Fig. 3A, B).

To confirm that Erastin and RSL3 induced cell death via ferroptosis in these cells, we used Ferrostatin-1, a specific ferroptosis inhibitor. Our results showed that cell viability was fully restored by Ferrostatin-1 in T98G cells, while in U251MG cells Ferrostatin-1 have not recovered cell death induced by Erastin (Fig. 3A, B). We further measured lipid peroxidation induction by Erastin and RSL3 treatment in both cell lines using BODIPY-C11 581/591 probe. The results demonstrated that T98G generates more lipid peroxidation upon Erastin and RSL3 treatment and this effect was successfully blocked by Ferrostatin-1 (Fig. 3C, D). In contrast, there was no increase in lipid peroxidation levels by ferroptosis inducers in U251MG cells. Next, we measured the GSH amount in both cell lines after Erastin treatment. Interestingly, we found that the blockage of system xc^−^ promoted GSH depletion only in the T98G cells (Fig. 3E). These results indicate a scenario of TMZ-resistant cell line vulnerability to pharmacological ferroptosis induction by GSH depletion, and TMZ-sensitive cell line resistance to ferroptosis through GSH synthesis maintenance.

### *NRF2* silencing promotes ferroptosis resistance through GSH regulation

**Fig. 4:**
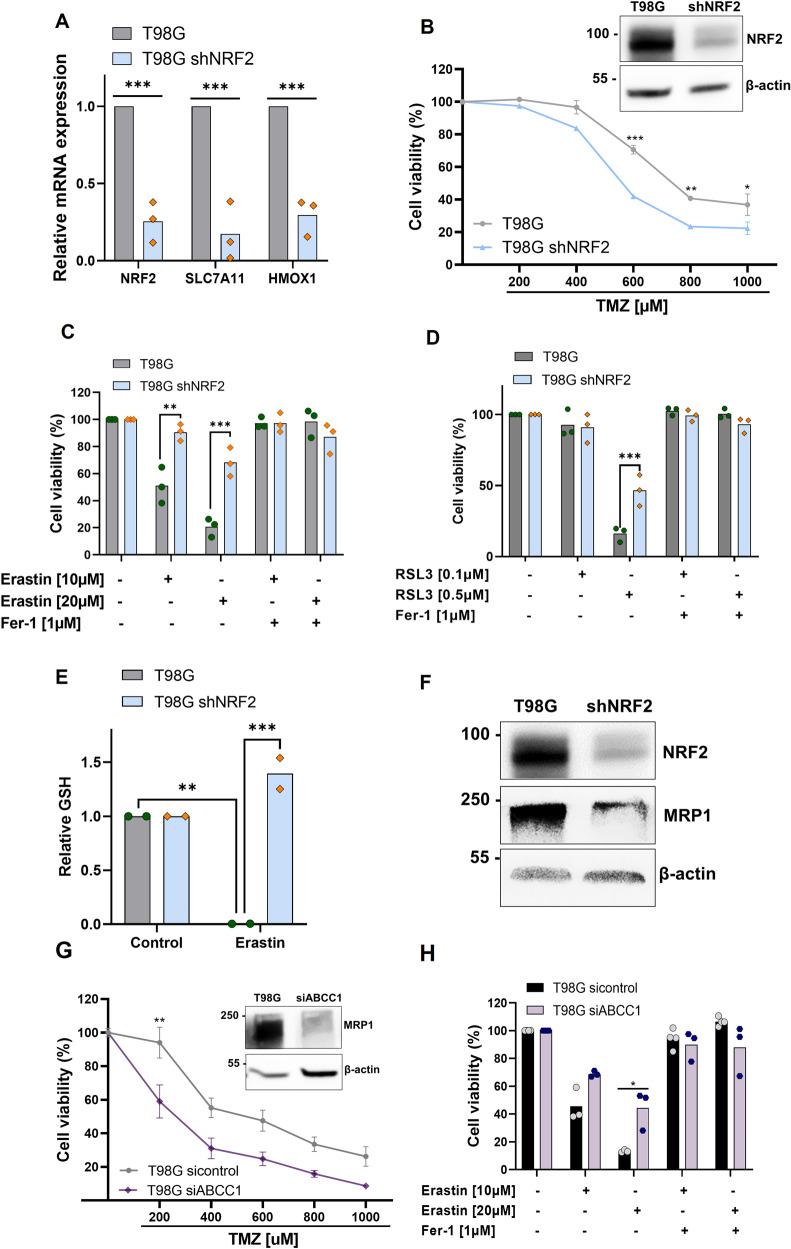
*NRF2* high expression promotes ferroptosis sensitivity through MRP1 regulation. **A** Quantitative real-time PCR analysis of NRF2, SLC7A11, and HMOX1 mRNA levels in T98G wildtype cell line and T98G transduced with sh*NRF2* lentivirus. **B** A dose-response curve of T98G and T98G-sh*NRF2* cell lines treated with increasing concentrations of TMZ (200–1000 μM) and analyzed 120 h after drug treatment measured by XTT assay/ NRF2 detection protein by western-blot in T98G and T98G-*NRF2* silenced cell. **C** Cells were treated with Erastin (10 and 20 μM) and Ferrostatin-1 (1 μM) for 72 h and viability was measured by XTT assay. **D** Cells were treated with RSL3 (0.1 and 0.5 μM) and Ferrostatin-1 (1 μM) for 72 h, and viability was measured by XTT assay. **E** Quantification of GSH intracellular levels after Erastin (20 μM) treatment for 24 h in T98G and T98G-sh*NRF2*. **F** NRF2 and MRP1 detection protein by western-blot in T98G and T98G-sh*NRF2*. **G** A dose-response curve of T98G and T98G si*ABCC1* cell lines treated with increasing concentrations of TMZ (200–1000 μM) and analyzed 120 h after drug treatment measured by XTT assay/ NRF2 and MRP1 detection protein by western-blot in T98G and T98G si*ABCC1*. **H** Cell viability analysis following Erastin treatment (10 and 20 μM) for 72 h in T98G wildtype and *ABCC1* silenced cells measured by XTT assay. Values are mean ± SEM of two or three independent experiments, ns = not statistically significant, *P < 0.05, **P < 0.01, ***P < 0.001, ****P < 0.0001. Each dot represents an independent experiment.

Next, we investigated if the high expression of *NRF2* could be related to ferroptosis sensitivity in T98G cells. In this regard, we established T98G-*NRF2* silenced cells (T98G-sh*NRF2*) using an shRNA lentiviral system. As shown in Fig. 4A, T98G-sh*NRF2* presented a significant reduction of mRNA expression of *NRF2* and its targets *SLC7A11* and *HMOX1*. Consistent with that, there was a significant decrease in NRF2 protein levels in T98G-sh*NRF2* and the silenced cells were significantly more sensitive to TMZ (Fig. 4B). Next, we examined cell viability upon treatment with ferroptosis inducers and we observed that T98G-sh*NRF2* cells showed greater resistance to ferroptosis compared to wild-type cells (Fig. 4C, D).

To investigate whether GSH depletion was associated with *NRF2* high expression in T98G cells, we treated cells with Erastin and quantified the GSH amount (Fig. 2E). We observed that *NRF2* silencing reversed the decrease of GSH levels in T98G, indicating that higher expression of *NRF2* could be contributing to the deficit in GSH amount in these cells.

### *ABCC1* high expression promotes ferroptosis in glioblastoma

Recent studies have shown that *ABCC1*/MRP1, a well-known *NRF2* target, can promote multidrug resistance through drug efflux and, at the same time, collaterally sensitize the cell to exogenous compounds [24, 33]. *ABCC1* was also described as a pro-ferroptotic gene through its capacity to generate GSH efflux and promote ferroptosis sensitivity in lung cancer cells [24]. Since our results suggest that *NRF2* high expression is involved both in T98G cell line drug resistance and ferroptosis sensitivity, we hypothesized that this dual role of *NRF2* is related to its target gene *ABCC1*, involved in collateral sensitivity and GSH regulation.

Thus, to explore whether *NRF2* promotes ferroptosis through *ABCC1*/MRP1, we first examined if high levels of *NRF2* were associated with an increase of *ABCC1*/MRP1 in the cell lines. As previously shown, the T98G cell line has a higher basal *NRF2* expression, and it also displayed higher mRNA levels of *ABCC1* and MRP1 protein levels when compared to U251MG (Fig. 2A, B). Accordingly, *NRF2* silencing leads to a substantial decrease in MRP1 protein levels (Fig. 4F), indicating MRP1 modulation by *NRF2* in these cells.

Given these results, we used small interfering RNA (siRNA) to silence *ABCC1* in T98G cells. As shown in Fig. 4G, T98G si*ABCC1* cells had a decrease in MRP1 protein levels, and they were more sensitive to TMZ treatment. Consistent with the results obtained in T98G-sh*NRF2* cells and confirming our hypotheses, *ABCC1* silencing promoted resistance to ferroptosis, as shown by the XTT assay (Fig. 4H). Thus, elevated *ABCC1* levels promotes collateral sensitivity in this glioblastoma cell line since it contributes to the GSH depletion when cells were exposed to blockage of system xc^-^ .

### U251MG cells are resistant to ferroptosis due to maintenance of GSH levels

**Fig. 5:**
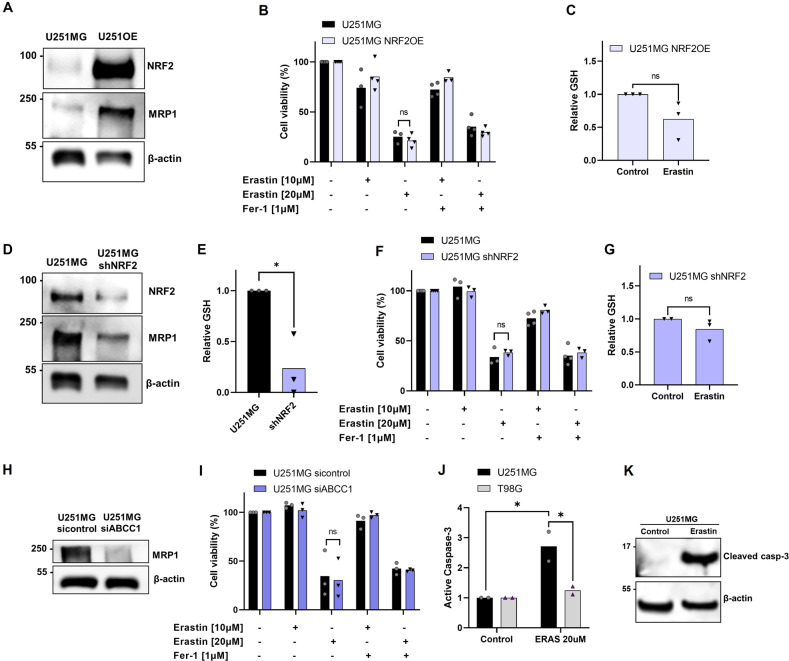
U251MG cells are resistant to ferroptosis. **A** NRF2 and MRP1 detection protein by western-blot in U251MG and U251MG *NRF2* OE. **B** Cell viability analysis following Erastin treatment (10 and 20 μM) and Ferrostatin-1 (1 μM) for 72 h in U251MG wildtype and *NRF2* overexpressed cells measured by XTT assay. **C** Quantification of GSH intracellular levels after Erastin (20 μM) treatment for 24 h in U251MG *NRF2* OE. **D** NRF2 and MRP1 detection protein by western-blot in U251MG and U251MG sh*NRF2*. **E** Quantification of basal GSH intracellular levels in U251MG and U251MG sh*NRF2*. **F** Cell viability analysis following Erastin treatment (10 and 20 μM) and Ferrostatin-1 (1 μM) for 72 h in U251MG wildtype and sh*NRF2* cells measured by XTT assay. **G** Quantification of GSH intracellular levels after Erastin (20 μM) treatment for 24 h in U251MG sh*NRF2*. **H** MRP1 detection protein by western-blot in U251MG sicontrol and U251MG si*ABCC1* cells. **I** Cell viability analysis following Erastin treatment (10 and 20 μM) and Ferrostatin-1 (1 μM) for 72 h in U251MG sicontrol and U251MG si*ABCC1* cells measured by XTT assay. **J** Flow cytometry analysis of the percentage of active caspase-3. **K** Western blot detection of cleaved caspase-3 in U251MG cells after treatment with Erastin (20 μM). Values are mean ± SEM of two or three independent experiments, ns = not statistically significant, *P < 0.05, **P < 0.01, ***P < 0.001, ****P < 0.0001. Each dot represents an independent experiment.

Since *NRF2* high expression have promoted ferroptosis sensitivity in T98G cells, we tested if overexpression of *NRF2* in U251MG could sensitize this cell line. The U251MG-*NRF2*OE cell line displayed a high expression of NRF2 and MRP1 protein (Fig. 5A). Then, we tested the vulnerability of U251MG *NRF2*OE cells to ferroptosis (Figs. 5B, S1A), and we observed no effect of sensitivity to ferroptosis in these cells compared to wild-type. Next, we investigated the levels of GSH after Erastin treatment in these cells (Fig. 5C). Our results showed that there was no change in GSH levels after system xc^-^ blockage when *NRF2* is upregulated, as well as seen in wild-type (Fig. 3E), indicating a possible mechanism of resistance of these cells by maintainance of GSH levels.

To better explore the role of *NRF2* in ferroptosis sensitivity of U251MG cells, we used *NRF2 or ABCC1* silenced U251MG cell line (Fig. 5D, H). The U251-shNRF2 presented significant lower levels of GSH in comparison to wild-type (Fig. 5E). The silencing of *NRF2* or *ABCC1* had no effect in ferroptosis sensitivity (Figs. 5F, I and S1B, S1C). Upon Erastin treatment, *NRF2* silencing was not capable of deplete the GSH levels (Fig. 5G). Since ferroptotic inducers failed to generate lipid peroxidation or cause change in GSH levels in U251MG cells (Fig. 3C–E), we infered that U251MG could escape from ferroptotic cell death by the compensatory mechanism of GSH synthesis.

Importantly, Ferrostatin-1 was not able to suppress ferroptosis in any analyzed cell lines (Figs. 3A, 5B, F, I). Recently, it has been shown that Erastin could be triggering another type of cell death, such as apoptosis [34]. With that in mind, we analyzed the caspase-3 levels, a hallmark of apoptosis, after Erastin treatment in both cell lines by flow cytometry. As shown in Fig. 5J, there was an increase in active caspase-3 after Erastin treatment in U251MG cells, but not in T98G. Western blot analyses were consistent with previous results (Fig. 5K), indicating higher levels of cleaved caspase-3 in U251MG after Erastin treatment. Thus, these results clearly indicate that Erastin induces apoptotic cell death in U251MG cells, but not in T98G.

### *ABCC1* expression is increased in high-grade glioma and is associated with worse patient outcomes

**Fig. 6:**
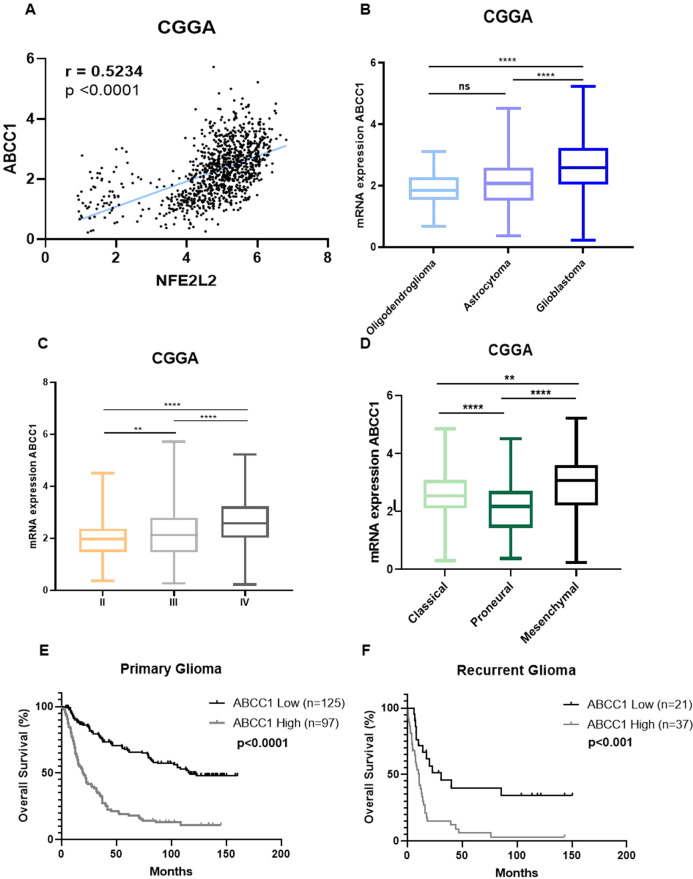
Gene expression analysis of glioma patients. **A** Correlation between *NRF2* and its target *ABCC1* in glioma patients. **B** ABCC1 mRNA expression in the CGGA cohort of patients stratified by histology; **C** grade; **D** and glioma subtype. **E** Kaplan–Meier curves showing overall survival of primary and **F** recurrent glioma patients from the CGGA cohort stratified according to ABBC1 expression. Patients were subgrouped into high *ABCC1* expression (above median) and low *ABCC1* expression (below median). ns = not statistically significant, *P < 0.05, **P < 0.01, ***P < 0.001, ****P < 0.0001.

As observed in the T98G cell line, the sensitivity to ferroptosis could be related to MRP1 high expression in glioblastoma TMZ-resistant cells. Thus, ferroptotic cell death could be a promising approach for sensitize those patients who are resistant to TMZ treatment and presented higher expression of MRP1. Once we demonstrated that *NRF2* has a positive correlation with *ABCC1* expression in vitro (Figs. 2A, B, 4F, and 5A, D), we next investigated whether the expression of those genes would present a similar pattern in glioma patients. Notably, we found a positive correlation between *NRF2* and *ABCC1* gene expression in three cohorts of glioma patients (CGGA, TCGA, and REMBRANDT) (Figs. 6A and S2A, S3A). Then, we explored *ABCC1* gene expression in human glioma tumor tissues. Our analyses revealed higher levels of *ABCC1* in glioblastoma patients compared to other glioma types (oligodendroglioma and astrocytoma) (Figs. 6B and S2B, S3B). Likewise, high grade glioma cases (according to WHO classification) displayed increased levels of *ABCC1* (Figs. 6C and S2C, S3C). Among the glioblastoma subtypes, the expression of *ABCC1* was elevated in the mesenchymal subtype (Figs. 6D and S2D, S3D).

Kaplan-Meier curves with the CGGA, TCGA, and REMBRANDT datasets showed that glioma patients with higher levels of *ABCC1* had a significantly shorter overall survival (OS) compared with patients with lower *ABCC1* levels (Figs. 6E and S2E, S3E). In the CGGA cohort of patients, for instance, we observed a 5-year OS of 18% versus 68% for primary glioma patients with higher *ABCC1* expression *versus* lower *ABBC1* expression, respectively (*p* < 0.0001) (Fig. 6E). Recurrent glioma patients with higher *ABCC1* also presented decreased OS (Fig. 6F). Considering only the patients who were treated with TMZ, higher *ABCC1* also associated with a significant reduction in survival (Fig. S4), suggesting a potential role of *ABCC1* in glioma drug resistance.

Patient overall survival was also estimated using a COX regression model. With a median follow-up time of 23.5 months, univariate analysis showed that high levels of *ABCC1* negatively impacted OS (*p* < 0.001). Multivariate analysis indicated that higher *ABCC1* was an independent prognostic factor for inferior OS in glioma (HR = 1.10, CI = 1.06–1.14), along with WHO grade and diagnosis age (all *p* < 0.05) (Table 1). A Bootstrap resampling procedure revealed the bias-independent accuracy of the *ABCC1* prediction and showed no overfitting in the cohort. Altogether, these results indicate that high *ABCC1* expression is related to higher tumor aggressiveness, chemotherapy resistance, and poorer overall survival in glioma, indicating an interesting scenario for ferroptosis induction.

**Table 1:** Univariate and multivariate analysis for OS of glioma patients from the CGGA cohort according to ABCC1 expression.

	Overall survival (*n* = 325)					
	Univariate analysis			Multivariate analysis		
Factor	HR^a^	95% C.I.	*P*	HR	95% C.I.	*P*
WHO Grade	4.673	3.489–6.259	<0.001	3.354	2.444–4.604	<0.001
Diagnosis age	1.033	1.02–1.044	<0.001	1.014	1.002–1.026	<0.022
ABCC1	**1.155**	**1.121–1.190**	**<0.001**	**1.104**	**1.067–1.143**	**<0.001**

^a^Statistically significant differences are highlighted in bold. Hazard ratios (HR) > 1 indicate that increasing values for continuous variable or the first factor for categorical variable has the poorer outcome. Grade was established according to 2016 WHO classification [1]. Diagnosis age and *ABCC1* expression were analyzed for continuous variation.

## Discussion

In the present work, we used two human glioblastoma cell lines (U251MG and T98G), which have already been shown to be TMZ-sensitive and TMZ-resistant, respectively [6, 35, 36]. TMZ can induce oxidative stress in glioblastoma cells, promoting protein damage and triggering oxidative cell death processes [37–39]. Therefore antioxidant pathways are related to preventing stress and protecting the cells against cell death [39]. In that sense, our group has previously shown that *NRF2* plays a central role in TMZ-resistance in glioblastoma cells both in vitro and in vivo [11, 40]. As mentioned before, T98G has *NRF2* gene amplification [31] and our data showed that this cell line has a greater level of GSH and lower intracellular ROS, indicating the protective role of *NRF2* in the T98G chemoresistance process. High levels of *NRF2* also could protect cells from ferroptosis through GSH and GPX4 upregulation [21]. However, our results unexpectedly demonstrated that TMZ-resistant cells with high levels of *NRF2* and GSH were extremely sensitive to ferroptosis. Moreover, *NRF2* disruption promoted resistance to ferroptosis.

**Fig. 7:**
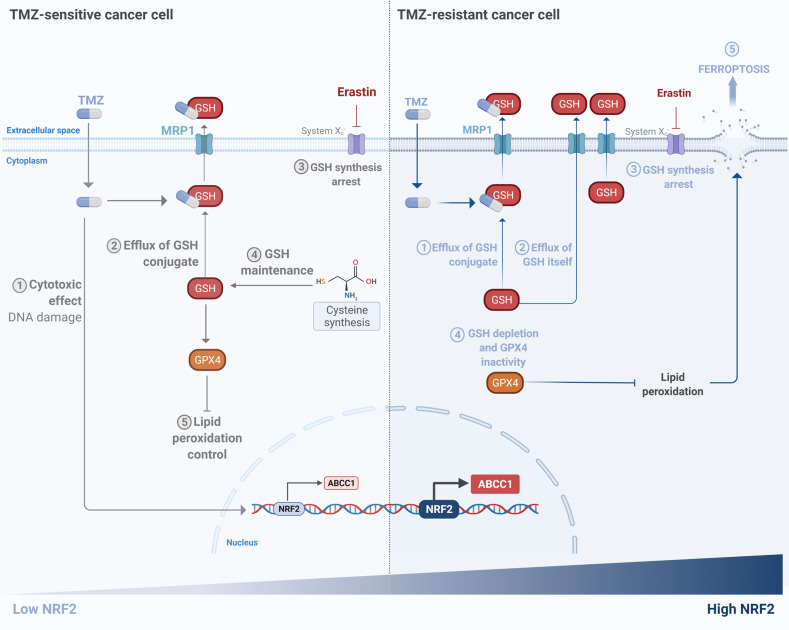
Schematic view of the mechanism of ferroptosis sensitivity in drug-resistant glioma cells. TMZ-resistant cells have higher expression of *NRF2* and, consequently, higher levels of *ABCC1*/MRP1. Upon treatment with TMZ, GSH mediates drug efflux by GSH-conjugate through MRP1 channels enhancing chemotherapy tolerance. Simultaneously, higher levels of MRP1 promote GSH efflux. Upon treatment with Erastin, there was a decrease in GSH synthesis, which is accentuated by GSH efflux through MRP1 promoting GPX4 inactivity and sensitizing these cells to ferroptosis. In contrast, TMZ-sensitive cells can obtain cysteine through compensatory mechanisms to generate GSH synthesis, thus they become resistant to ferroptosis. Created with BioRender.com.

As we have shown, *NRF2* regulates the expression of the multidrug resistance-associated proteins 1 (MRP1/*ABCC1*), which has an important role in drug resistance through its capacity of mediating the cellular efflux of GSH-drug conjugates [33]. MRP1 also exports GSH itself [41, 42], thus high activation of this gene could sensitize cells to ferroptosis, since GSH is crucial to prevent iron-dependent cell death [24, 43]. Here, we demonstrated that cells with higher expression of *NRF2*, and consequently, higher *ABCC1* expression, had a significant depletion of GSH levels when cystine uptake was blocked by Erastin, due to high MRP1 activity, leading to ferroptotic cell death. This indicates that MRP1 activity results in the phenomenon known as collateral sensitivity (CS) in glioblastoma cells. In that sense, glioblastoma cells with high expression of *NRF2* could evade cell death induced by TMZ through the GSH-drug efflux by high levels of MRP1, becoming chemoresistant. These elevated amounts of MRP1 could simultaneously collaterally sensitize TMZ-resistant cells to ferroptosis upon Erastin treatment, as it is schematically shown in Fig. 7.

While our work supports a recently described mechanism of ferroptosis modulation by *NRF2*, it demonstrates, for the first time, the effect in glioblastoma cells: TMZ-resistant glioblastomas cells show higher vulnerability to ferroptosis induction due to high expression of *NRF2* and its target *ABCC1*. Since high MRP1 expression is crucial to eliminating TMZ-resistant glioma cells through ferroptosis induction in vitro, exploiting collateral sensitivity by ferroptosis induction could be the Achilles’ heel to reverse drug resistance in glioblastoma.

Thus, even in *NRF2* high expression background, glioblastoma cells could be sensitive to ferroptosis. Although *NRF2* regulates the transcription of genes involved in GSH synthesis, and could protect some cancer cells from ferroptosis [21–23], here we provide evidence that *NRF2* expression is a poor predictor of ferroptotic sensitivity for glioblastoma cells since the role of *NRF2* in ferroptosis could be actually determined by MRP1, which counterbalances the protective effects of GSH synthesis by *NRF2*. Indeed, it was recently demonstrated that *NRF2* expression, as well as basal intracellular total GSH, are not sufficient to predict the sensitivity to ferroptosis in HAP1 cells [24]. Also, mRNA *NRF2* levels were poor predictors to determine ferroptosis sensitivity in NSCLC, glioblastoma, osteosarcoma, and fibrosarcoma cells [24]. NRF2 high expression in T98G cells could also indicate a state of permanent oxidative stress that needs high antioxidant performance, which could easily lead to the triggering of ferroptosis when a pro-ferroptotic gene is upregulated [44]. Accordingly, it was reported that NRF2 supports melanoma dedifferentiation, which induces ferroptosis [44], and drug-resistant cells were more responsive to ferroptosis induction [45, 46].

Furthermore, our results using U251MG (low levels of NRF2) also demonstrated an interesting scenario of ferroptosis resistance in glioblastoma cells independent of NRF2 expression, in which *NRF2* modulation and ferroptosis inhibitors were not sufficient to reverse the cell death caused by ferroptosis inducers. Interestingly, in this cell line the blockage of the system xc^−^ did not affect GSH levels, indicating that these cells could obtain cysteine from a compensatory mechanism. Indeed, it has been demonstrated that cysteine obtained from methionine by transsulfuration pathway contributes to GSH synthesis, even upon system xc^−^ blockage, leading to tumor developing and conferring resistance to Erastin in ovarian cancer cells [47–49]. Since we observed cleaved caspase-3 upon Erastin treatment, we concluded that Erastin failed to induce ferroptosis, but actually induced apoptotic cell death in these cells. In fact, it was demonstrated that Erastin can potentially activate VDAC channel [50], which results in mitochondrial disruption [51] and lead to apoptotic cell death in gastric [52] and colorectal cancer cells [34]. Overall, our results indicate that in TMZ-sensitive cells the maintenance of GSH synthesis when cystine uptake is inhibited by Erastin, prevents ferroptosis. Likewise, GSH depletion was sufficient to promote ferroptosis in TMZ-resistant cells, indicating that NRF2 role in ferroptosis could be determined not by intracellular GSH basal levels, but by the intracellular GSH modulation after treatment.

Finally, high *ABCC1* expression was associated with tumor severity and worse patient outcomes both in primary and recurrent glioma. Also, *ABCC1* gene expression is an independent prognostic factor in glioma patients, corroborating the need for a more effective treatment for this group of patients. In fact, high MRP1 expression promotes chemoresistance [53, 54] and leads to a poor clinical outcome in patients with neuroblastoma [55]. Thus, finding a pharmacological strategy for sensitizing *NRF2* high-expression tumors, such as T98G cell line, is a huge gain for patients who are not benefited from standard chemotherapeutic treatments.

Altogether, our data suggest an interesting approach for improving the chemotherapeutic treatment of TMZ-resistant tumors through ferroptosis induction mediated by GSH depletion, which requires further investigation in vivo and pre-clinical studies. At the same time, it demonstrates an important mechanism of resistance to ferroptosis of TMZ-sensitive cells through GSH synthesis upon system xc^−^ blockage.

## Material and methods

### Cell lines and culture conditions

Certified human glioma cell lines U251MG and T98G were kindly provided by Prof. Bernd Kaina, Germany. Both cell lines were recently authenticated by STR profiling and they tested negative for mycoplasma contamination. They were routinely cultured in DMEM (Invitrogen, Life Technologies, Carlsbad, CA, USA) or Opti-MEM medium supplemented with 10 and 5% FTS (fetal calf serum; Cultilab, Campinas, SP, Brazil) respectively and 1% antibiotic-antimycotic at 37 °C in a humidified, 5% CO_2_ atmosphere.

### Chemicals and reagents

Erastin and temozolomide were purchased from Cayman Chemical Company (Michigan, USA) and were dissolved in DMSO under sterile conditions to a concentration of 1 and 50 mM, respectively. 1S,3R-RSL3 (RSL3) and Ferrostatin-1 were purchased from Sigma-Aldrich (Taufkirchen, Germany) and were dissolved in DMSO at a concentration of 1 mM.

### Cell survival analysis

Cell survival was measured by Cell Proliferation Kit II (XTT) (Roche, Basel, Switzerland). 1 × 10^4^ cells were plated in 24-well plate and treated with TMZ and ferroptosis modulators for 72 or 120 h. After that, the cells were washed with phosphate-buffered saline (PBS) followed by incubation with XTT reagent kit as recommended by the manufacturer’s instructions.

### RT-qPCR

Total RNA extraction was performed by TRIzol Reagent (Invitrogen) as described in the manufacturer’s guideline and followed by DNase (Promega, Madison, WI, USA) treatment. cDNA was prepared using a High-Capacity cDNA Reverse Transcription kit (Applied Biosystems, Life Technologies). Quantitative PCR reactions were prepared with 6 μL of SYBR Green Master Mix (Applied Biosystems), 150 or 200 nM of forward and reverse primers, nuclease-free water, and 3 μL of diluted cDNA. Primer sequences for NRF2 (Fwd: 5′-AAACCAGTGGATCTGCCAAC-3′/Rev: 5′-TCTACAAACGGGAATGTCTGC-3′), SCL7A11 (Fwd: 5′-GCAAGCACACTCCTCTACCA-3′/Rev: 5′-AGCCCAATAAAAAGCCACCT-3′), HMOX1 (Fwd: 5′-AACTTTCAGAAGGGCCAGGT-3′/Rev: 5′-GTAGACAGGGGCGAAGACTG-3′), ABCC1 (Fwd: 5′- AGTGGAACCCCTCTCTGTTTAAG-3′) /Rev: 5′-CCTGATAGCTCTTGGTCTTCATC-3′), GAPDH (Fwd: 5′-ACCCACTCCTCCACCTTTGA-3′/Rev: 5′-CTGTTGCTGTAGCCAAATTCGT-3′), HPRT (Fwd: 5′-GAACGTCTTGCTCGAGATGTGA-3/Rev: 5′-TCCAGCAGGTCAGCAAAGAAT-3′). qPCR was carried out using the 7500 Real-Time PCR System (Applied Biosystems), relative transcript levels were calculated using the ΔΔCT method and normalized to the housekeeping genes GAPDH or HPRT.

### Clonogenic assay

Glioblastoma cells (1 × 10^3^) were seeded into 35-mm dishes or 6-well and treated with different concentrations of TMZ (5, 10, and 20 μM). After 10 days of treatment, cells were washed with PBS, fixed with 4 % paraformaldehyde, and stained with crystal violet. The colonies were photographed and manually counted.

### Flow cytometry for cell cycle, γH2AX, and caspase-3 analysis

8 × 10^4^ cells were plated in a 12-well plate. After treatment with TMZ (100 μM), Erastin, RSL3, and Ferrostatin-1 for 24, 48, and 72 h, cells were collected and fixed with 1 % formaldehyde and 70% ethanol. Ethanol-fixed cells were blocked, permeabilized, and incubated overnight at 4 °C with a primary mouse monoclonal antibody to γH2AX (Ser-139) (Upstate Biotechnology, Lake Placid, NY, USA) diluted 1:500, or mouse anti-active caspase 3 (BD, Pharmigen, San Diego, CA, USA) diluted 1:50 for 2 h at room temperature. Samples stained with γH2AX were incubated with anti-mouse FITC secondary antibody (Sigma-Aldrich) diluted 1:200 for 2 h at room temperature and then stained with (PI) at room temperature for 1 h in PBS containing 20 μg/ml PI (Sigma–Aldrich), 200 μg/ml RNase A and 0.1% Triton X-100. Analysis was performed with Guava EasyCyte Plus System flow cytometer (Guava Technologies, Hayward, CA, EUA) and the percentage of γH2AX positive cells, cell cycle analysis and caspase-3 analysis were calculated using the CytoSoft 5.3 software (Millipore, Billerica, MA, USA).

### Fluorescence-activated cell analysis

1 × 10^5^ cells were seeded in 12-wells plates and treated with TMZ (100 μM), Erastin, RSL3 (1 μM), and Ferrostatin-1 (1 μM) for 24 h. Briefly, cells were collected, washed with PBS and re-suspended in 10 μM DCFDA (2′,7′-dichlorofluorescein diacetate, Invitrogen) or 2 μM BODIPY C11 (581/591) (Invitrogen) probe diluted in PBS. After 30 min of incubation, analysis was performed with Guava EasyCyte or BD LSRFortessa flow cytometers, and analyses were carried out with FlowJo software (BD Biosciences). For microscopic analysis, 1 × 10^4^ cells were grown in six-well plates and stained with DCFDA or BODIPY-C11 for 30 min, then the pictures were taken by Inverted Microscope Axio Observer Z1 Zeiss. All imaging acquisition parameters were kept constant for each sample.

### Western Blot

Proteins were extracted from cell pellets lysed and quantified using the Pierce BCA Protein Assay kit (Thermo Scientific, Rockford, IL, EUA). After that, proteins were separated by electrophoresis on an SDS-polyacrylamide gel and transferred to a nitrocellulose membrane (GE Healthcare, Waukesha, WI, USA). Membranes were blocked for 1 h in 5% (w/v) milk powder in PBS and incubated overnight at 4 °C with primary antibody against anti-NRF2 (1:500) (#sc-722 Santa Cruz Biotechnology, Santa Cruz, CA, USA), anti-MRP1 (1:1000) (#ab3368 Abcam, Cambridge, UK), anti-caspase 3 cleaved (1:200) (#ab2302 Abcam), and anti-βactin (1:1000) (#4967 Cell Signaling). Then, membranes were incubated with the correspondent secondary antibody and a chemiluminescent HRP substrate (Merck Millipore. Burlington, MA, USA) was used to develop the membranes. Each blot was performed twice. The full and uncropped western blots can be found in Supplemental Material.

### Glutathione quantification

Intracellular GSH levels were quantified using the GSH/GSSG Ratio Detection Assay Kit (Fluorometric - Green) (abcam), following the manufacturer’s instructions. Briefly, 1 × 10^6^ cells were plated in a 60 mm plate and treated with the compound of interest. After 24 h, cells were collected and washed with cold PBS 1× and PBS 0.5% NP-40. After, samples were centrifugated and the supernatant was collected. We removed the enzymes from the samples by following the deproteinization protocol. Next, we prepared the Thiol Green Solution (1:50) and mixed it with the samples, after 30 min we measured the fluorescence (490/520 nm).

### Database analysis

RNA-seq expression data, clinical and molecular information of glioma patient samples were obtained from the GlioVis data portal for visualization and analysis of brain tumor expression datasets (http://gliovis.bioinfo.cnio.es/) and CGGA (Chinese Glioma Genoma Atlas) [56]. Clinical data is described in Table S1. For *NFE2L2* (*NRF2*) and *ABCC1* expression, survival, and correlation analysis, results were extracted from the CGGA (*n* = 325, batch 2), TCGA (https://portal.gdc.cancer.gov) (*n* = 664), and REMBRANDT (*n* = 397) cohort. Analysis of the differences in overall survival between two groups (*ABCC1* Low and *ABCC1* High) was performed using Kaplan-Meier curves and the log-rank test. For CGGA cohort, the median of *ABCC1* expression of all 325 samples (5.09) was used as the cutoff point. For TCGA and REMBRANDT studies, optimal cutoff points were designated by the GlioVis database and corresponded to 9.27 and median (8.21), respectively. Correlation analysis was performed with Pearson. Cox regression model was used to estimate patient overall survival (OS) in the CGGA cohort. The stepwise process of selection was used for multivariate analysis. OS was defined as the time between the date of sampling and the date of death (for deceased patients). For internal data validation, the Bootstrap Resampling tool was used [57].

### Statistical analysis

Statical analysis was performed by SPSS (IBM, NY, EUA) and GraphPad Prism 8 (GraphPad Software Inc., CA, USA). All results in vitro were represented as the mean ± S.E.M. of at least two or three independent experiments, each performed in duplicate or triplicate. Each graph shows independent data points representing each experiment. Statistical significance among data sets was compared with unpaired two-tailed Student’s t-test or two-way ANOVA (when comparing more than two groups) followed by Bonferroni multiple comparisons post-testing (ns = not statistically significant, *p < 0.05, **p < 0.01, ***p < 0.001, ****p < 0.0001). For database analysis *p* < 0.05 was considered statistically significant.

## Supplementary information

Supplementary Files

Reproducibility Checklist

Original Western Blots

Original Data File

Authors agreement

## Data Availability

The datasets generated and/or analyzed during the current study are available from the corresponding author on reasonable request.
