# Supplementary material for: High levels of NRF2 sensitize temozolomide-resistant glioblastoma cells to ferroptosis via ABCC1/MRP1 upregulation
Source: Cell Death Dis. 2022 Jul 8;13(7):591. doi: 10.1038/s41419-022-05044-9 (PMC9270336; doi:10.1038/s41419-022-05044-9)
Supplement: Supplementary file 1 — Supplementary Files [file 41419_2022_5044_MOESM1_ESM.docx]

**SUPPLEMENTARY FILES**

**
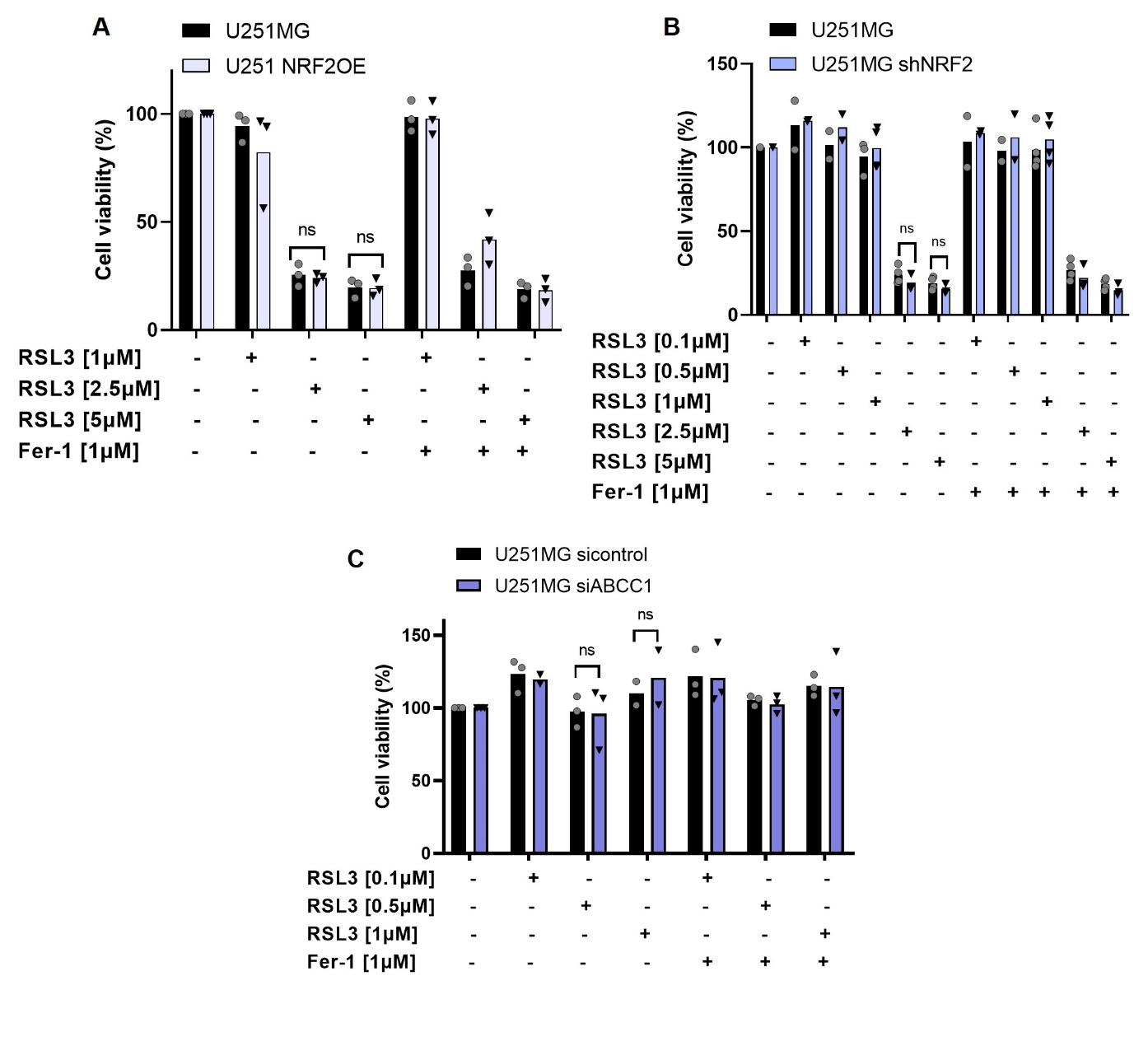
Supplemental Figure 1:**

**Supplemental figure 1 (S1): Analysis of U251MG sensitivity to RSL3. A)** Cell viability analysis following RSL3 treatment (1, 2.5, 5μM) and ferrostatin-1 (1 μM) for 72 h in U251MG wildtype and NRF2 overexpressed cells measured by XTT assay. **B)** Cell viability analysis following RSL3 treatment (1, 2.5, 5μM) and ferrostatin-1 (1 μM) for 72 h in U251MG and U251MG shNRF2 cells measured by XTT assay. **C)** Cell viability analysis following RSL3 treatment (0.1, 0.5, 1μM) and ferrostatin-1 (1 μM) for 72 h in U251MG and U251MG shNRF2 cells measured by XTT assay. Values are mean ± SEM of three independent experiments, ns = not statistically significant, *P< 0.05, **P< 0.01, ***P< 0.001, ****P<0.0001. Each dot represents an independent experiment.

**Supplemental Figure 2:**

**
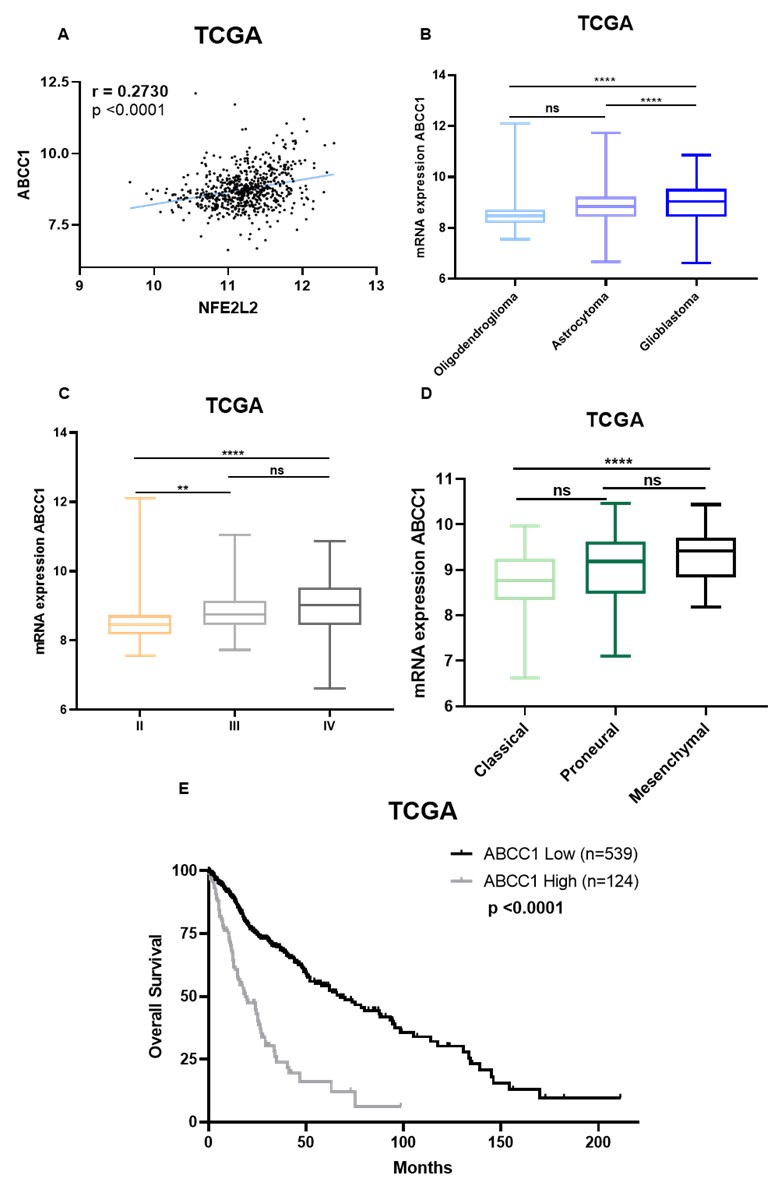
**

**Supplemental figure 2 (S2): Gene expression analysis of glioma patients in TCGA.**

**A)** Correlation between NRF2 and its target ABCC1 in glioma patients. **B)** ABCC1 mRNA expression in the CGGA cohort of patients stratified by histology; **C)** grade; **D)** and glioma subtype. **E)** Kaplan–Meier curves showing overall survival of patients from the TCGA cohort stratified according with ABBC1 expression. Patients were subgrouped into high ABCC1 expression (above 9.24) and low ABCC1 expression (below 9.24). The optimal cutoff point was designated by GlioVis database (9.24) (GlioVis:http://gliovis.bioinfo.cnio.es). ns = not statistically significant, *P< 0.05, **P< 0.01, ***P< 0.001, ****P<0.0001.

**Supplemental Figure 3:**


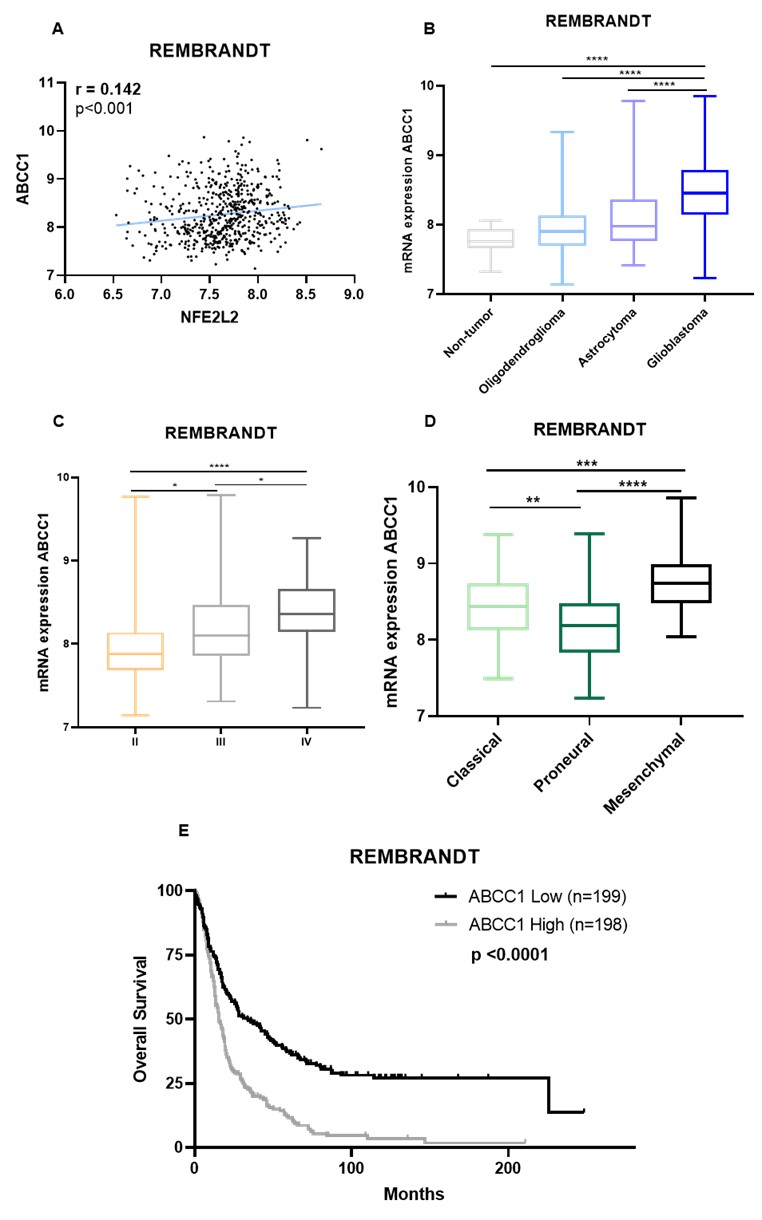


**Supplemental figure 3 (S3): Gene expression analysis of glioma patients in REMBRANDT. A)** Correlation between NRF2 and its target ABCC1 in glioma patients. **B)** ABCC1 mRNA expression in the CGGA cohort of patients stratified by histology; **C)** grade; **D)** and glioma subtype from. **E)** Kaplan–Meier curves showing overall survival of patients from the REMBRANDT cohort stratified according with ABBC1 expression. Patients were subgrouped into high ABCC1 expression (above median) and low ABCC1 expression (below median). The optimal cutoff point was designated by GlioVis database (median) (GlioVis:http://gliovis.bioinfo.cnio.es). ns = not statistically significant, *P< 0.05, **P< 0.01, ***P< 0.001, ****P<0.0001.

**Supplemental Figure 4:**

**
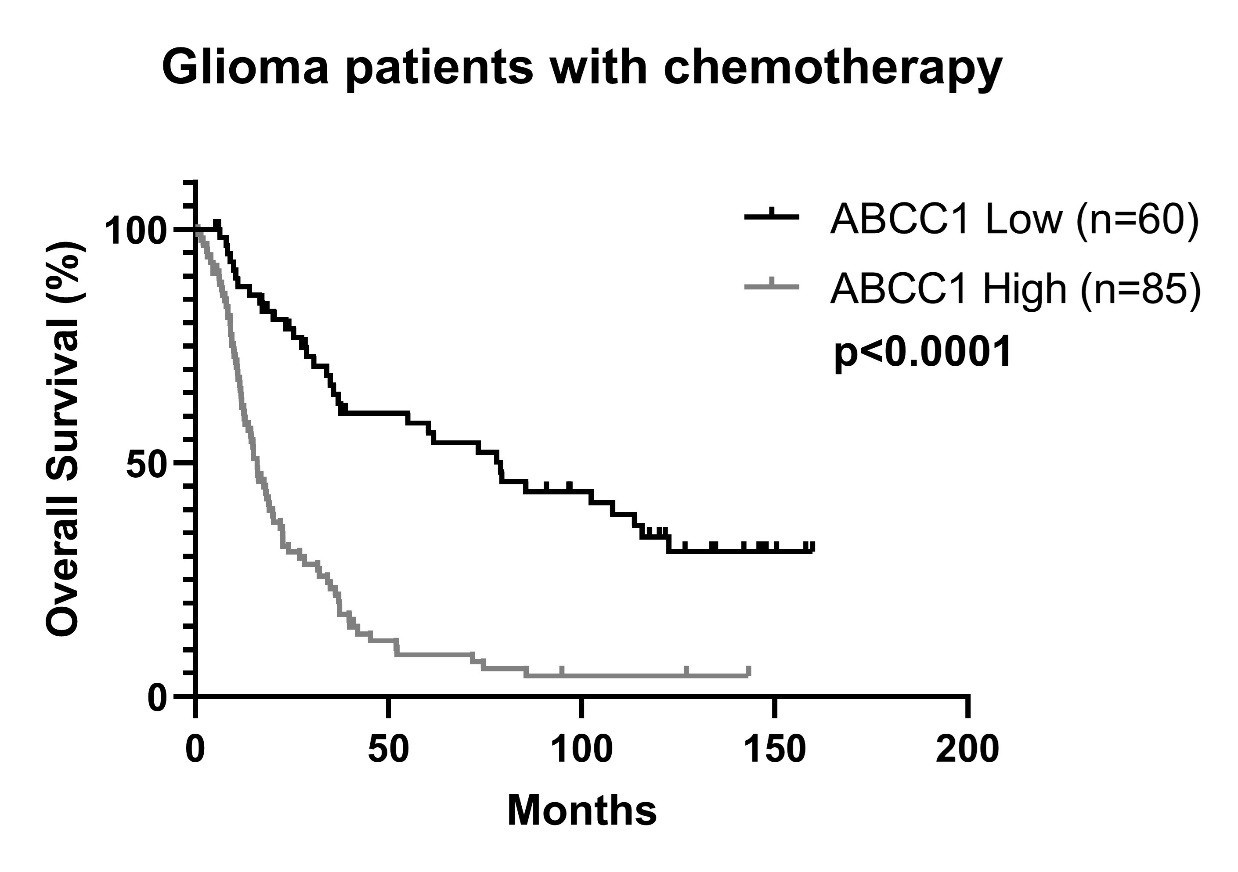
**

**Supplemental figure 4 (S4): Survival analysis in CGGA dataset.** Kaplan–Meier curves representing survival of patients with low vs high expression of ABCC1 in glioma patients with chemotherapy.

| Characteristics | CGGA | TCGA | REMBRANDT |
| --- | --- | --- | --- |
| **Total of cases** | **325** | **664** | **397** |
| **Gender** |  |  |  |
| Male | 203 | 353 | - |
| Female | 122 | 254 | - |
| N/A | - | 57 | - |
| **Age (Years)** |  |  |  |
| <40 | 182 | - | - |
| >40 | 143 | - | - |
| N/A | - | - | - |
| **Grade (WHO)** |  |  |  |
| II | 103 | 225 | 70 |
| III | 79 | 243 | 69 |
| IV | 139 | 149 | 106 |
| N/A | 4 | 47 | 152 |
| **IDH Status** |  |  |  |
| Mutation | 175 | 425 | - |
| Wildtype | 149 | 232 | - |
| N/A | 1 | 7 | - |
| **1p19q Status** |  |  |  |
| Non-codel | 250 | - | - |
| Codel | 67 | - | - |
| N/A | 8 | - | - |

**Supplemental Table 1:**

**Supplemental Table (S1):** Characteristics of patient samples in CGGA, TCGA and REMBRANDT database.

*N/A: not available
