# Supplementary material for: High levels of NRF2 sensitize temozolomide-resistant glioblastoma cells to ferroptosis via ABCC1/MRP1 upregulation
Source: Cell Death Dis. 2022 Jul 8;13(7):591. doi: 10.1038/s41419-022-05044-9 (PMC9270336; doi:10.1038/s41419-022-05044-9)

ORIGINAL WESTERN BLOTS

Note: NRS = Not related sample.

Figure 2B:

NRF2:

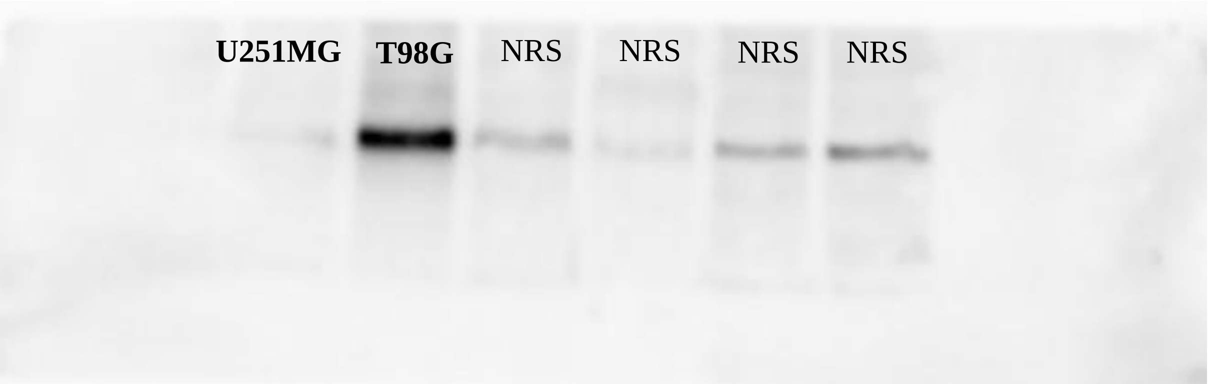

b-actin of NRF2 membrane:

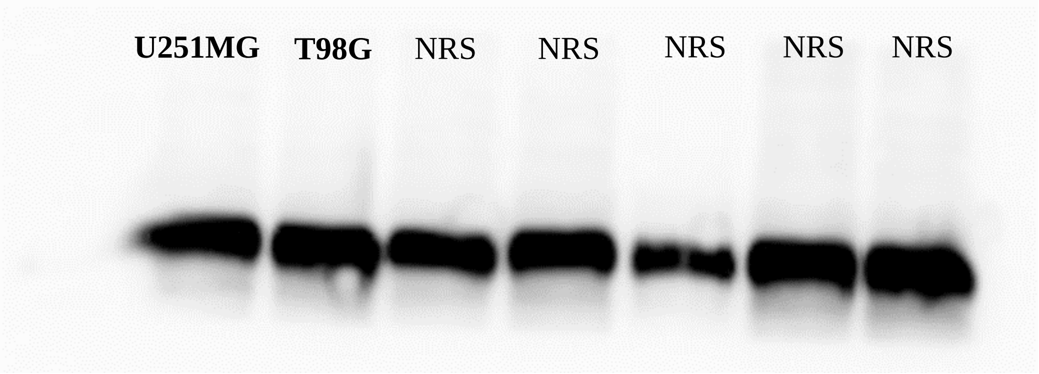

MRP1:

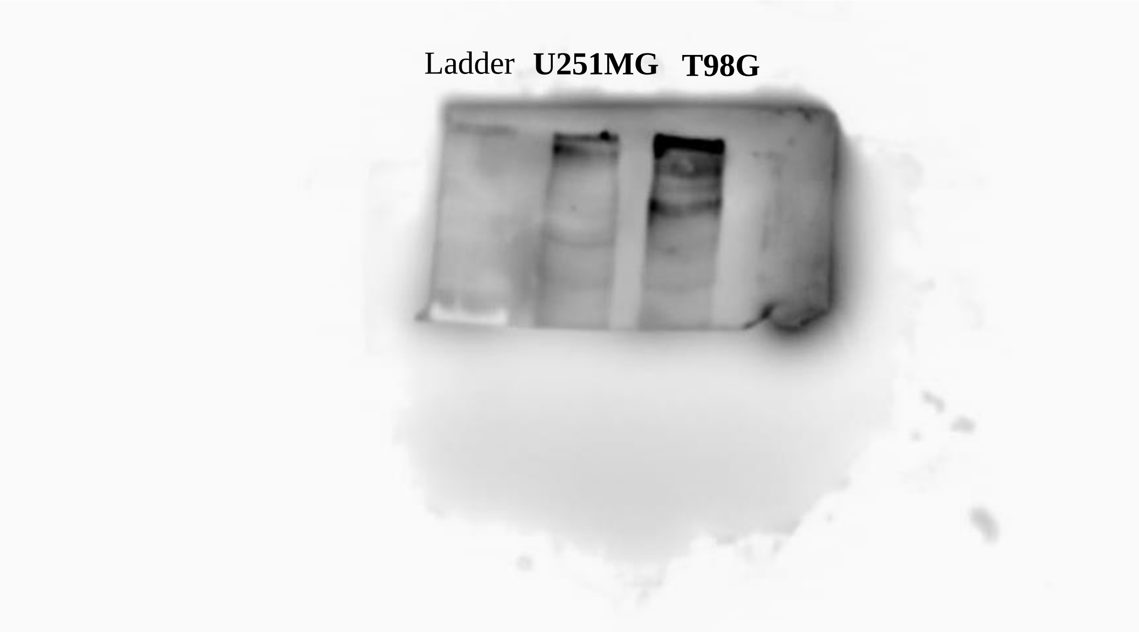

b-actin of MRP1 membrane:

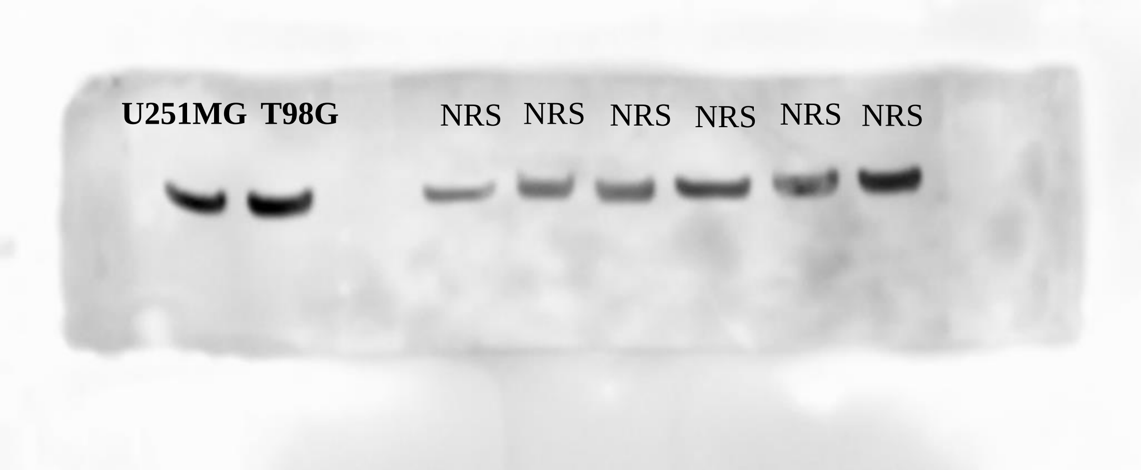

Figure 4B:

NRF2:

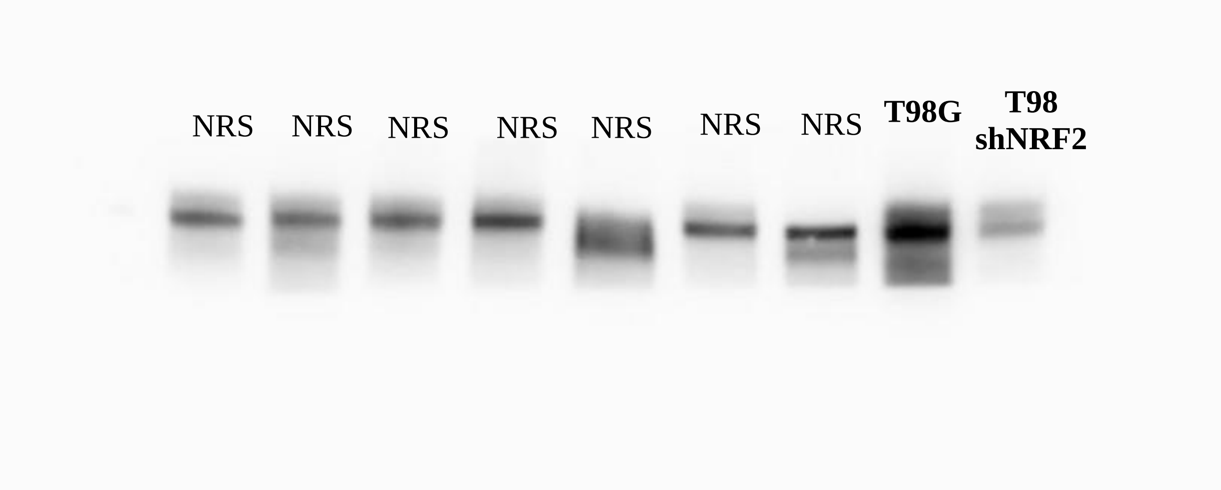

b-actin:

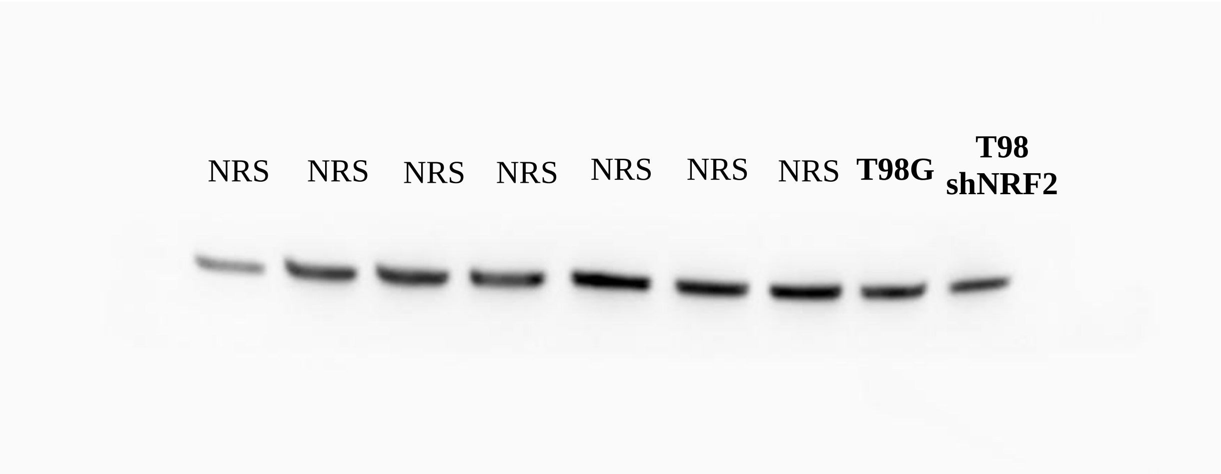

Figure 4F:

MRP1:

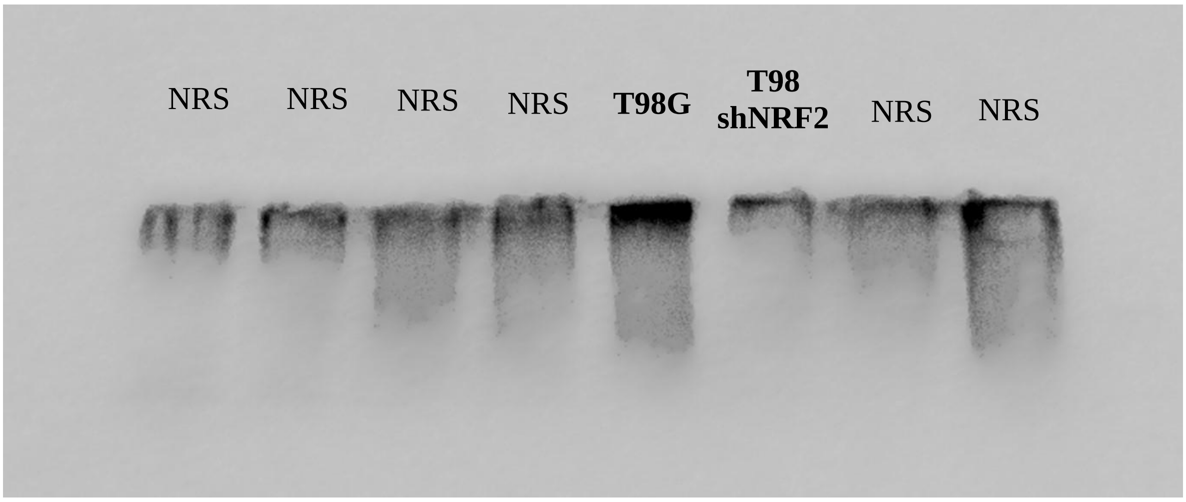

b-actin:

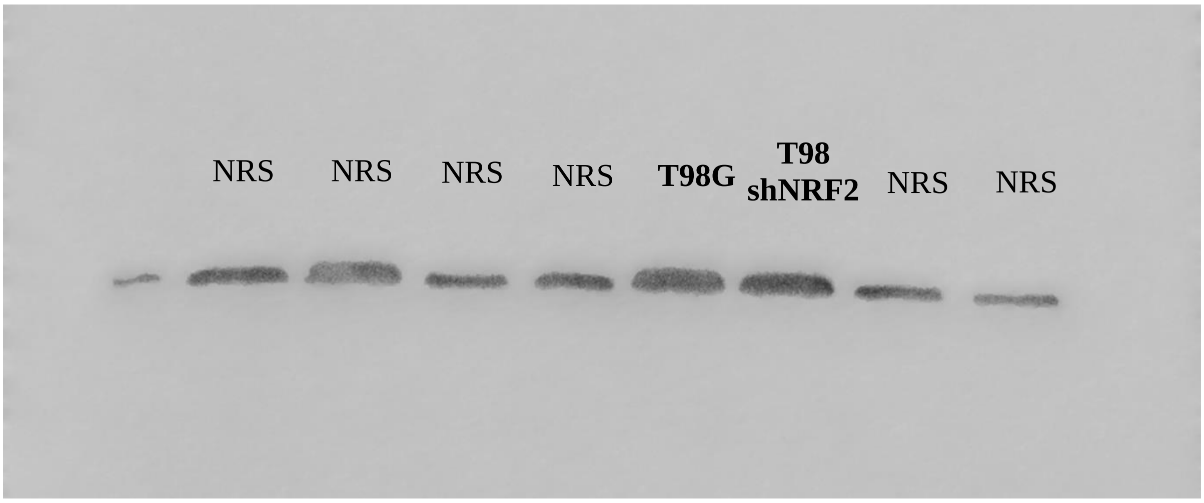

Figure 4G:

MRP1:

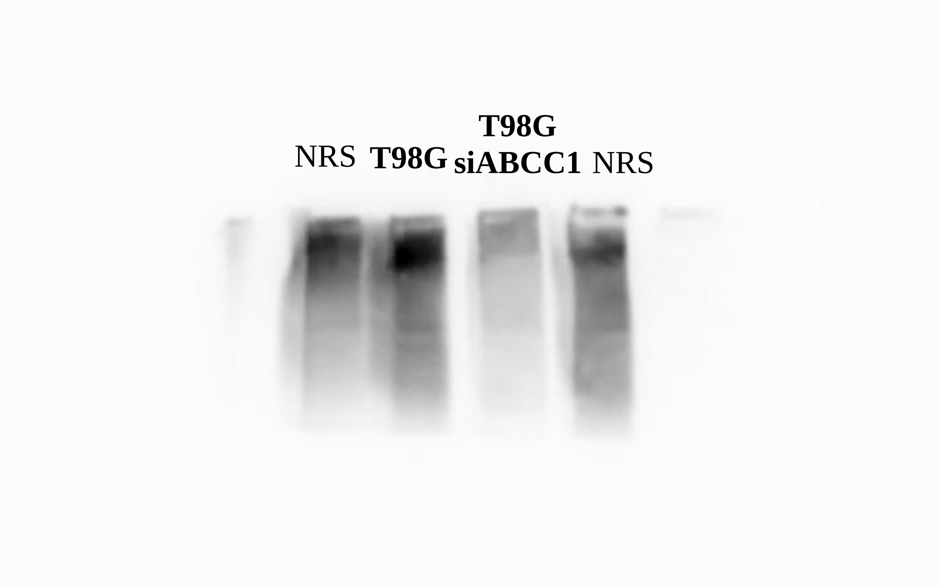

b-actin:

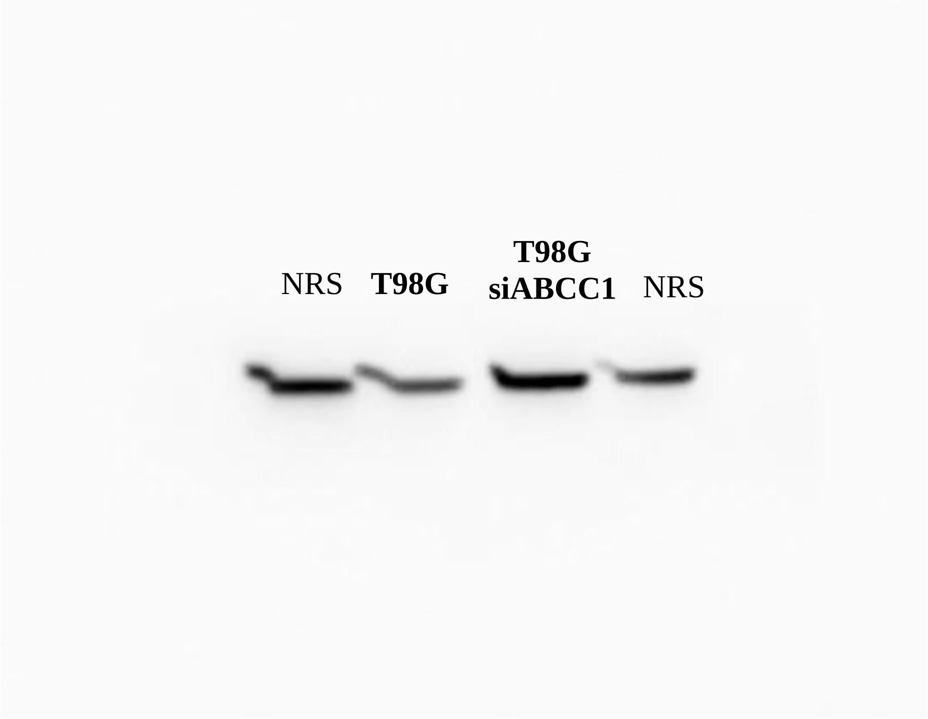

Figure 5A:

NRF2:

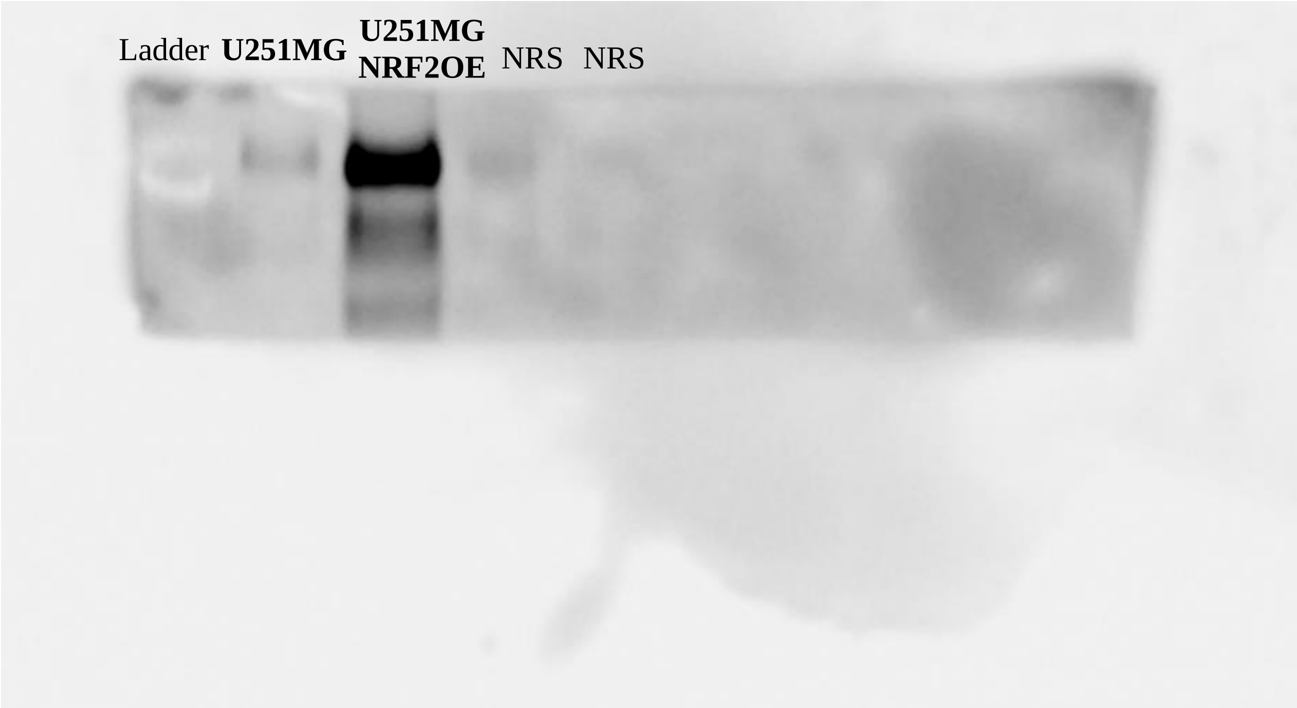

MRP1:

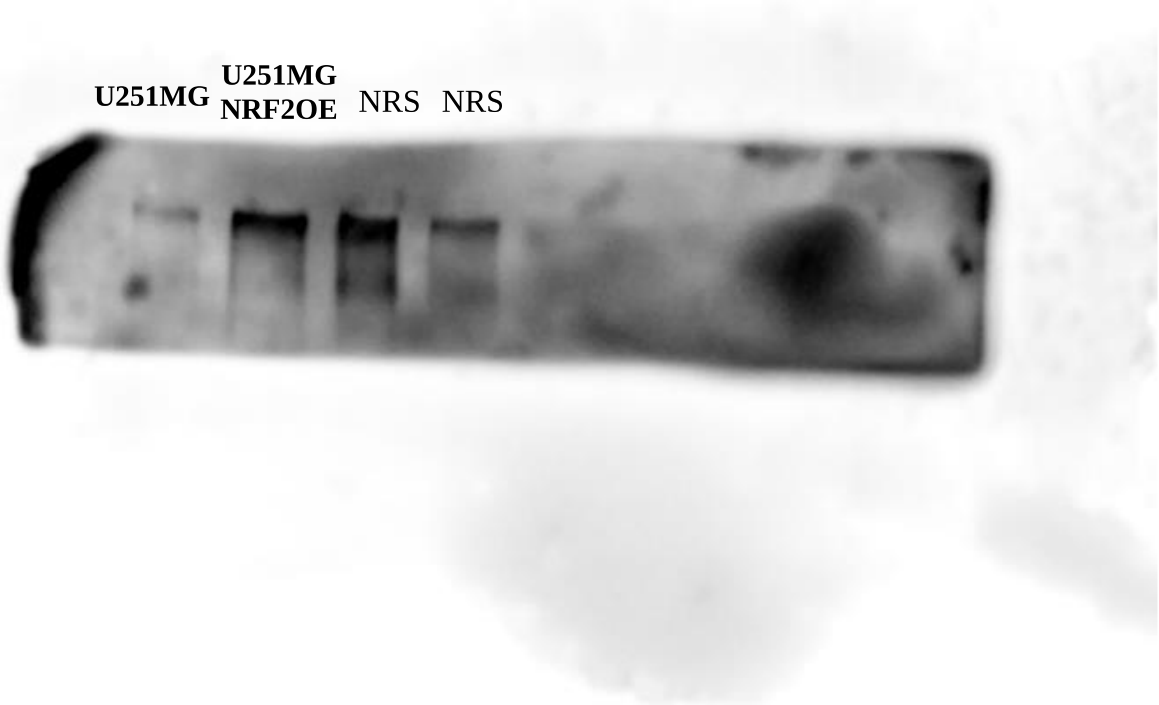

b-actin of NRF2 and MRP1 membrane:

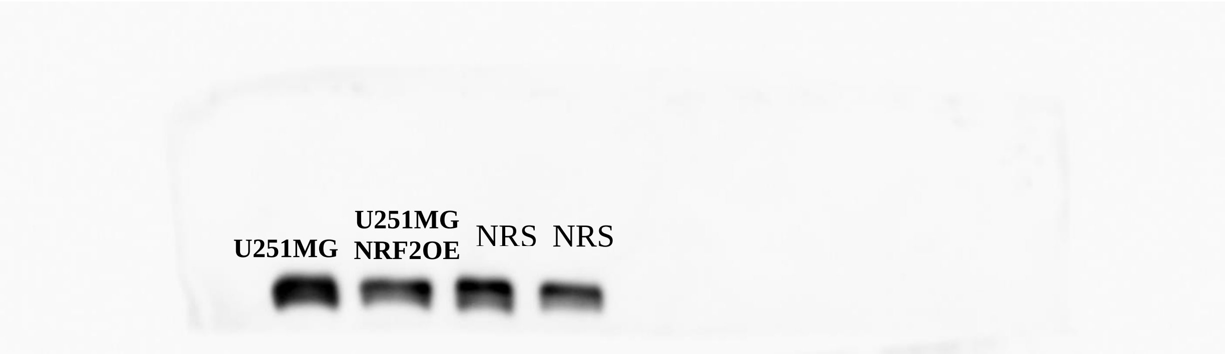

Figure 5D:

NRF2:

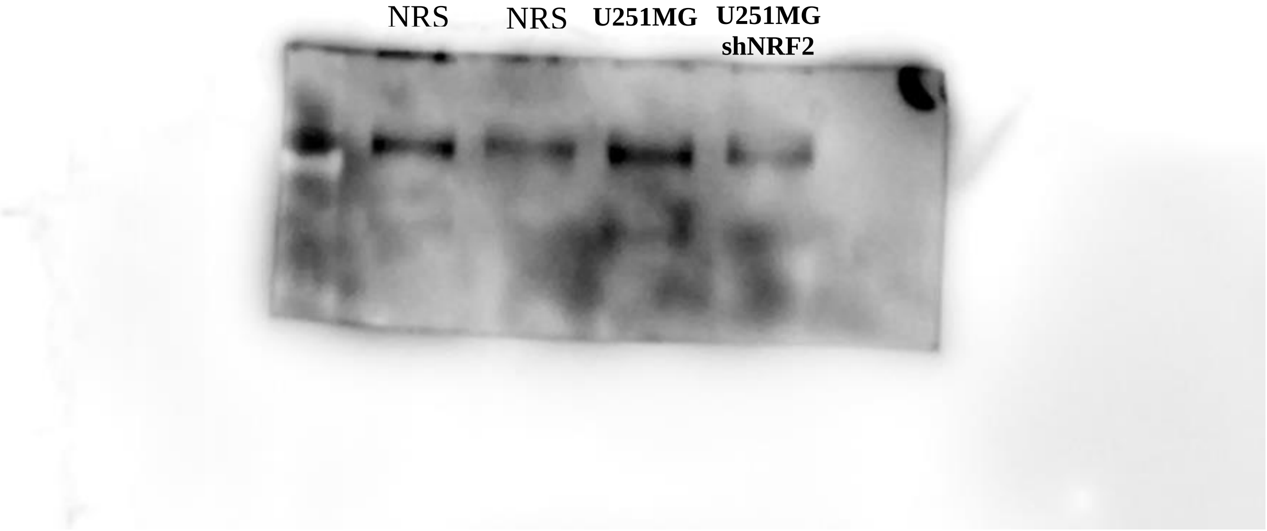

Ponceau of NRF2 membrane:

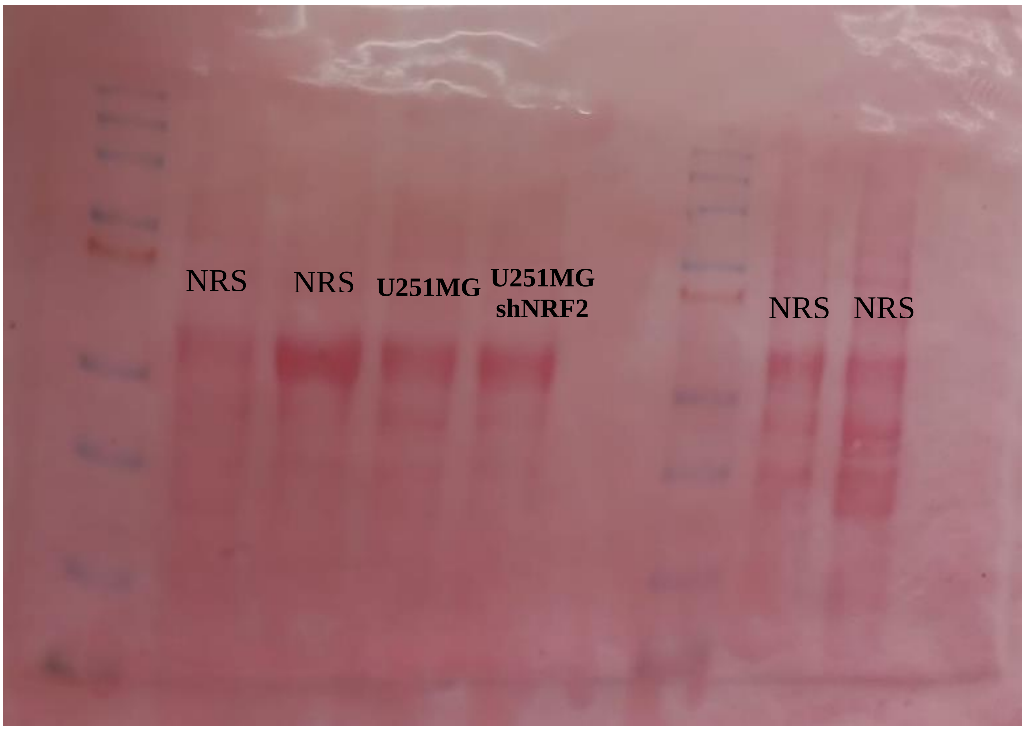

MRP1:

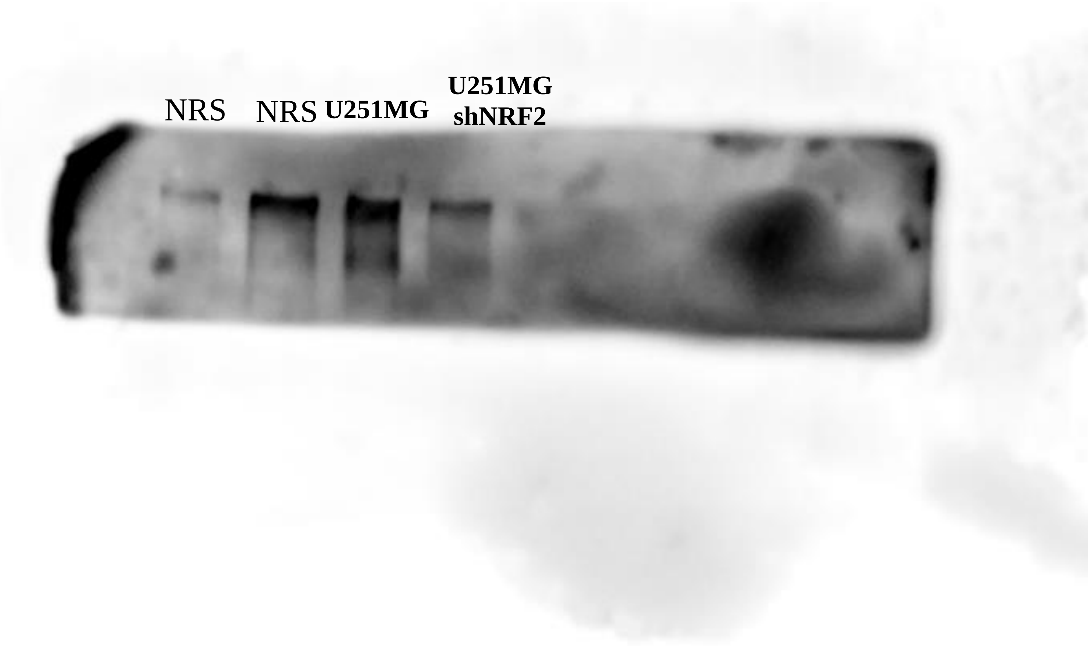

b-actin of MRP1 membrane:

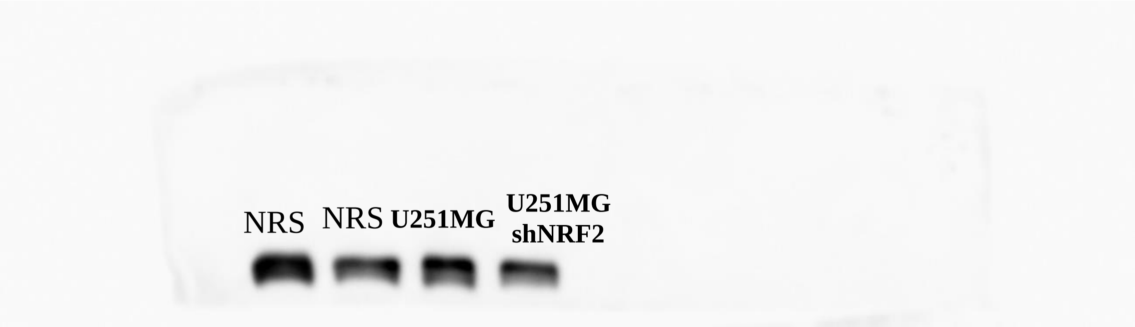

Figure H:

MRP1:

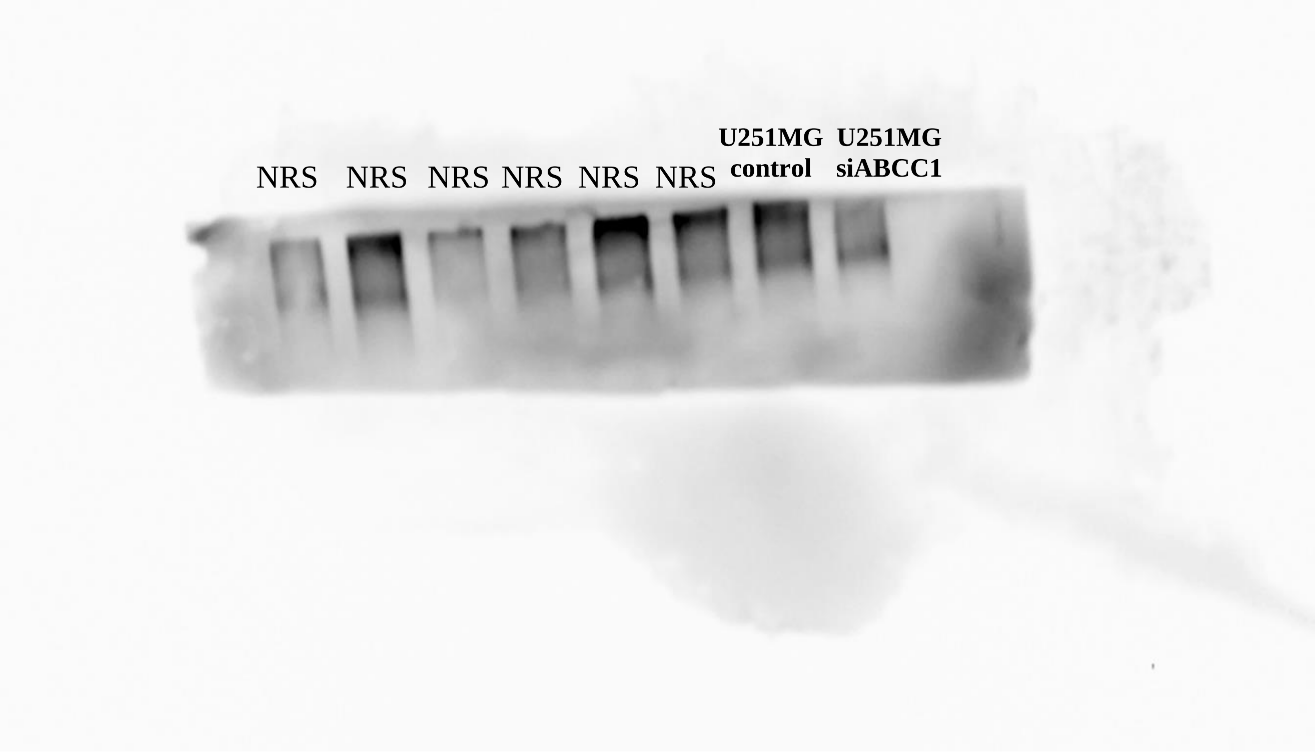

b-actin:

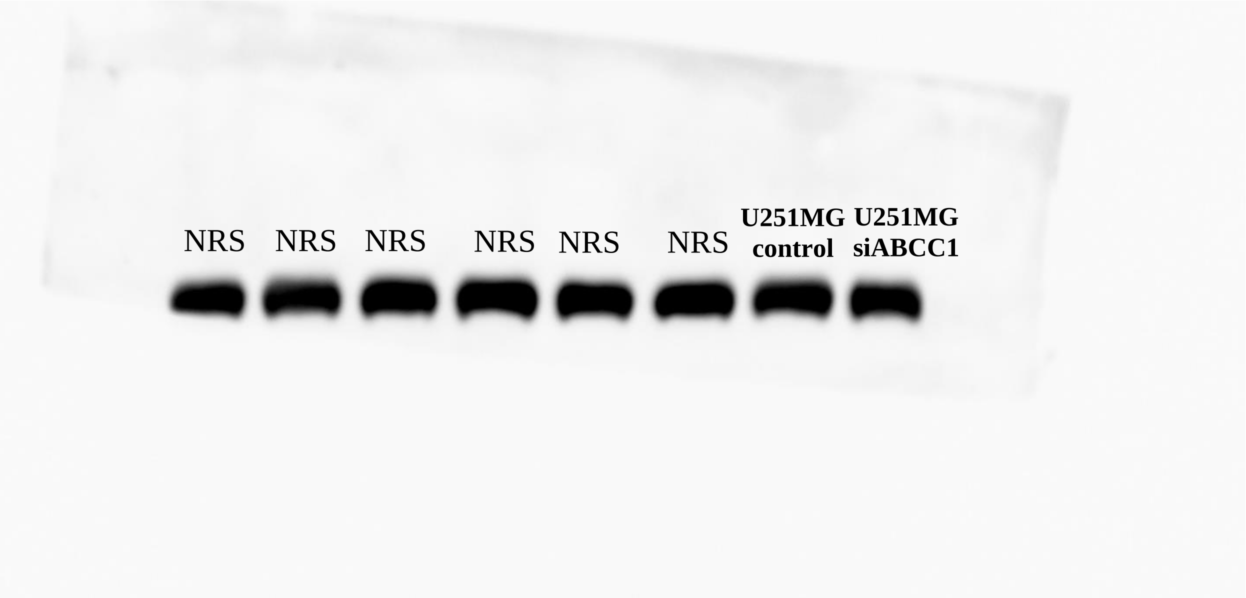

Figure 5K:

Caspase-3 cleaved:

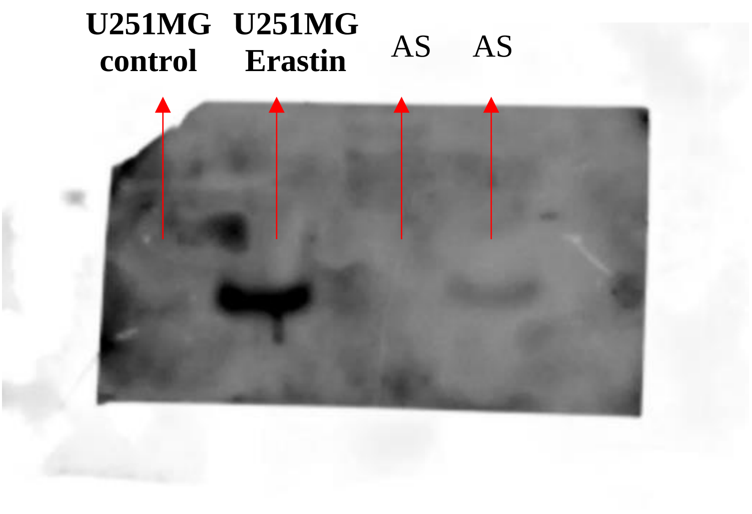

b-actin:

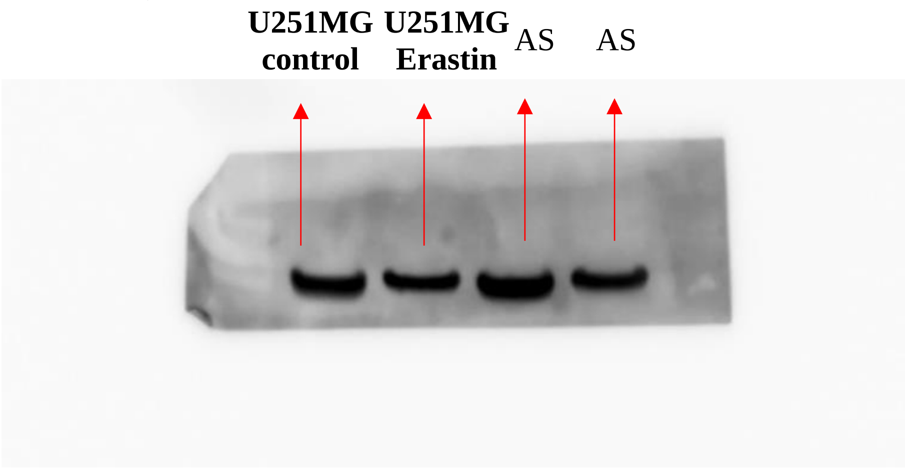

Supplement: Supplementary file 3 — Original Western Blots [file 41419_2022_5044_MOESM3_ESM.pdf]
