# Supplementary material for: High levels of NRF2 sensitize temozolomide-resistant glioblastoma cells to ferroptosis via ABCC1/MRP1 upregulation
Source: Cell Death Dis. 2022 Jul 8;13(7):591. doi: 10.1038/s41419-022-05044-9 (PMC9270336; doi:10.1038/s41419-022-05044-9)

**ORIGINAL DATA FILES**

**Figure 1A:**


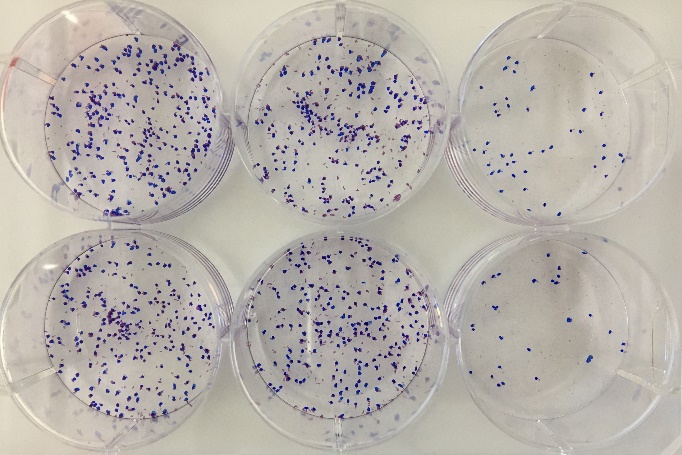
U251MG:


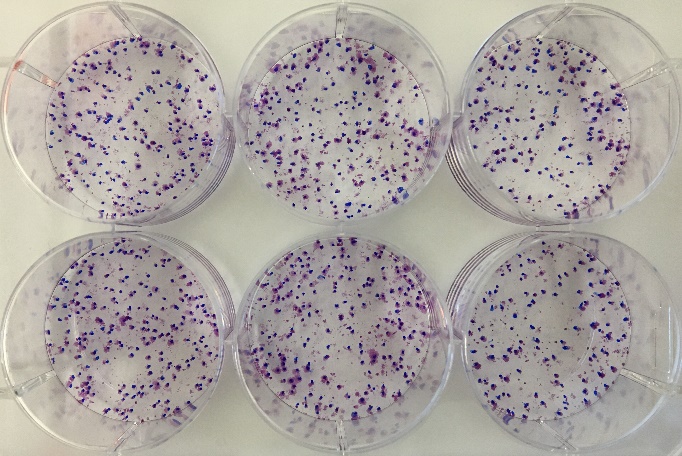
T98G:

**Figure 2E:**

U251MG BRIGHT:


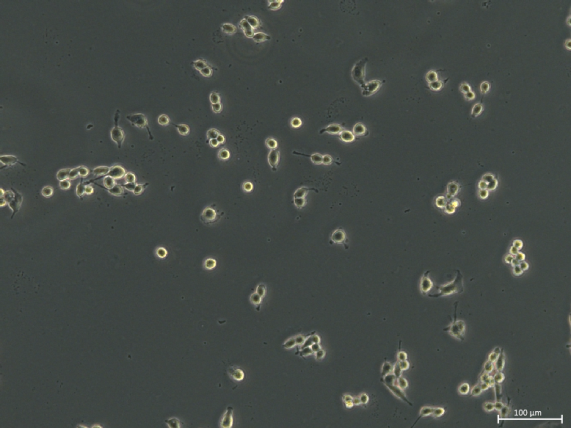


U251MG DCFDA


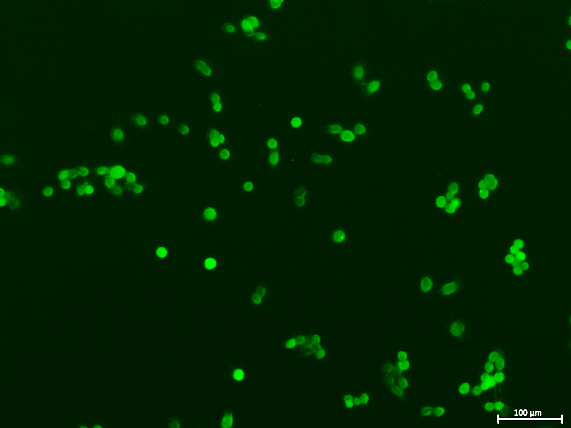


T98G BRIGHT:


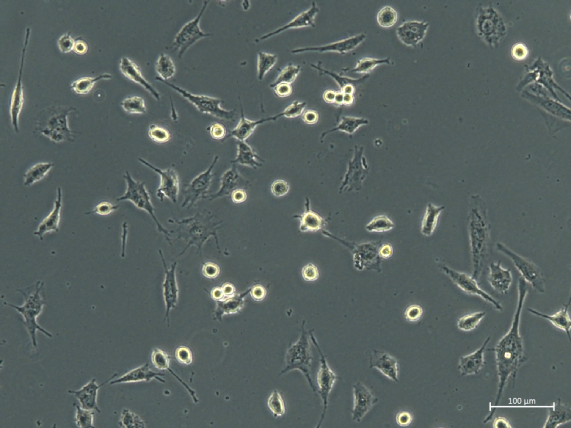


T98G DCFDA:


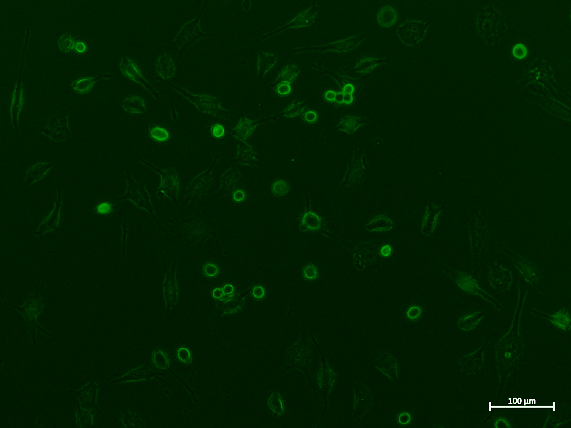


**Figure 2H:**

U251MG BRIGHT:


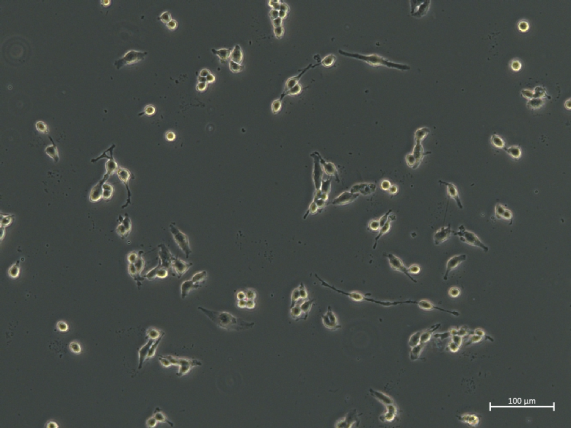


U251MG BODIPY-C11:


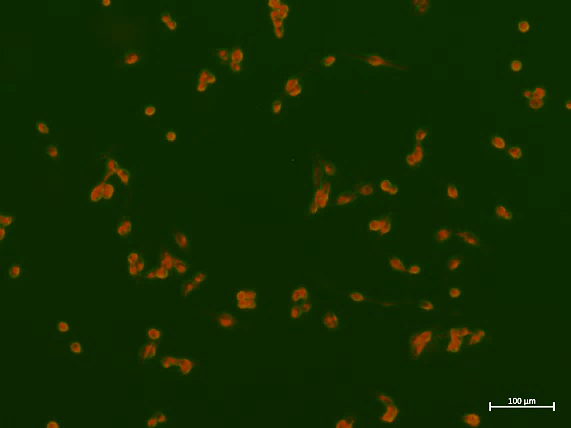


T98G BRIGHT:


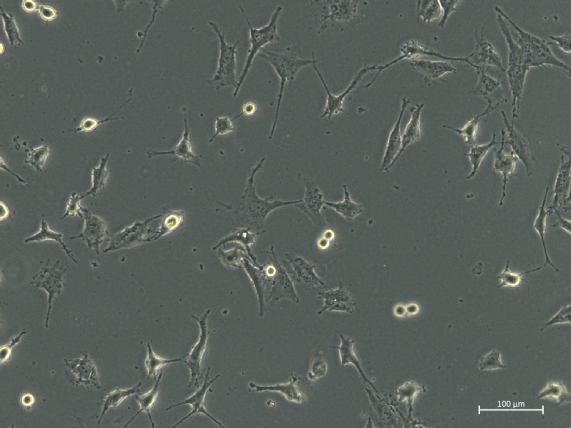


T98G BODIPY C-11:


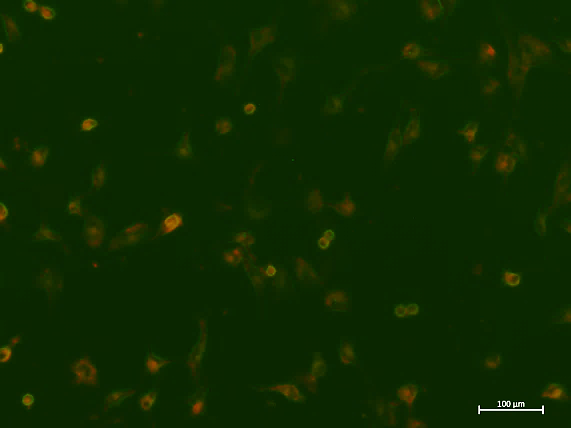

Supplement: Supplementary file 4 — Original Data File [file 41419_2022_5044_MOESM4_ESM.docx]
