# Supplementary material for: High levels of NRF2 sensitize temozolomide-resistant glioblastoma cells to ferroptosis via ABCC1/MRP1 upregulation
Source: Cell Death Dis. 2022 Jul 8;13(7):591. doi: 10.1038/s41419-022-05044-9 (PMC9270336; doi:10.1038/s41419-022-05044-9)
Supplement: Supplementary file 5 — Authors agreement [file 41419_2022_5044_MOESM5_ESM.pdf]

UNIFESP

CLARISSA RIBEIRO REILY ROCHA <clarissa.rocha@unifesp.br>

CDDIS-21-5003RRR Initial Quality Check

13 mensagens

---

cddisease@springernature.com <cddisease@springernature.com> 23 de junho de 2022 12:53
Responder a: cddisease@springernature.com
Para: clarissa.rocha@unifesp.br

Dear Dr Rocha,

In checking in your manuscript submitted to Cell Death & Disease it has come to our attention that the following must be addressed before we can proceed.

1. It has come to our attention that your most recent author list differs from the one in your original submission. We find that the order has changed.

Please request agreement from all authors including additions and deletions, these can be collected in the following way:

Email your co-authors with the change, and ask them to reply to your email confirming that they agree to these changes. Once you have collected these replies, please combine all of the co-authors' email responses in one document and upload this file to your submission.

2. Please make sure all figures and tables are cited within the article file in numerical order.

3. Please ensure your table legends are included in the table file only and not the article file.

4. All Articles must be accompanied by a Reproducibility checklist (http://www.nature.com/authors/policies/checklist_AJ.pdf) ensuring you do not leave any of the boxes blank, please write 'n/a' instead if they do not apply.

Your paper is now available for you to edit, you may access via the following link:

https://mts-cddis.nature.com/cgi-bin/main.plex?el=A6CA3Wco6D4CBoQ2F7A9ftdk09FiWeJq4crXo6kR9YgZ

(Press/Click on the above link to be automatically sent to the web page.)

Please make the correction(s) as specified above and resubmit your paper following the same steps as before.

If you have any questions please feel free to contact us.

Sincerely,

Editorial Office
Cell Death & Disease
cddisease@springernature.com

This email has been sent through the NPG Manuscript Tracking System NY-610A-NPG&MTS

Confidentiality Statement:

This e-mail is confidential and subject to copyright. Any unauthorised use or disclosure of its contents is prohibited. If you have received this email in error please notify our Manuscript Tracking System Helpdesk team at http://platformsupport.nature.com .

Details of the confidentiality and pre-publicity policy may be found here http://www.nature.com/authors/policies/confidentiality.html

Privacy Policy | Update Profile

CLARISSA RIBEIRO REILY ROCHA <clarissa.rocha@unifesp.br> 23 de junho de 2022 13:11
Para: "izadora.souza@unifesp.br" <izadora.souza@unifesp.br>, Linda Seregni <linda.seregni@unifesp.br>, Camila Guedes <cguedes16@hotmail.com>, Matheus Molina <matheusmolina95@gmail.com>, MARINA ANDRADE TOMAZ <marina.tomaz@unifesp.br>, Bruna Contieri <brunacontieri@hotmail.com>, Marcela Latancia <marcela.latancia@gmail.com>, Bruna Maldonado <brumaldonado@gmail.com>, Davi Mendes <davimendes4@gmail.com>, MARIANA LAZARINI <lazarini@unifesp.br>, Luciana Gomes <luciana.gomes@butantan.gov.br>

Dear co-authors

I am contacting you all to call your attention to two changes that we want to make in our manuscript.

First, in the latest version of our manuscript we chanced the order of the authors and now it is:
Ms Izadora Souza , Ms Linda Seregni Monteiro , Ms Camila Banca Guedes , Mr Matheus Molina Silva , Ms Marina Tomaz-Andrade , Ms Bruna Contieri , Ms Marcela Teatin Latancia , Dr Davi Mendes , Ms Bruna Felício Milazzotto Maldonado Porchia Ribeiro , Mariana Lazarini , Dr Luciana Rodrigues Gome, Dr. Clarissa Ribeiro Reily Rocha

Second, we noticed that, unfortunately, the name of one of the authors was misspelled. So it is written **Marina Tomaz-Andrade**, but should it be **Marina Andrade-Tomaz**

Thus, I would like to ask you to reply to this email confirming that you agree to both changes.
Please do reply as soon as possible
Best regards
Clarissa
[Texto das mensagens anteriores oculto]
--
Dra. Clarissa Ribeiro Reily Rocha
Coordenadora do Programa de Pós-graduação em Medicina (Hematologia e Oncologia)
Departamento de Oncologia Clínica e Experimental
Escola Paulista de Medicina - UNIFESP
Rua Dr. Diogo de Faria, 824, 5o Andar
Laboratório de Biologia Celular e Molecular
Vila Clementino, 04037-003, São Paulo - SP
Tel: 55 11 988355110

Marcela Latancia <marcela.latancia@gmail.com> 23 de junho de 2022 13:15
Para: CLARISSA RIBEIRO REILY ROCHA <clarissa.rocha@unifesp.br>
Cc: izadora.souza@unifesp.br, Linda Seregni <linda.seregni@unifesp.br>, Camila Guedes <cguedes16@hotmail.com>, Matheus Molina <matheusmolina95@gmail.com>, MARINA ANDRADE TOMAZ <marina.tomaz@unifesp.br>, Bruna Contieri <brunacontieri@hotmail.com>, Bruna Maldonado <brumaldonado@gmail.com>, Davi Mendes <davimendes4@gmail.com>, MARIANA LAZARINI <lazarini@unifesp.br>, Luciana Gomes <luciana.gomes@butantan.gov.br>

Dear Clarissa and Izadora

Congratulations again! I agree with the changes.

Best regards
Marcela Teatin Latancia

> On Jun 23, 2022, at 12:11 PM, CLARISSA RIBEIRO REILY ROCHA <clarissa.rocha@unifesp.br> wrote:

> [Texto das mensagens anteriores oculto]
> ----
> A Universidade Federal de São Paulo segue a política de e-mail Institucional disponível em https://sti.unifesp.br/documentos/termos-de-uso-do-email

---

Izadora Souza <izadora.souza@unifesp.br> 23 de junho de 2022 13:27
Para: CLARISSA RIBEIRO REILY ROCHA <clarissa.rocha@unifesp.br>

I do agree with the changes.

Izadora de Souza

[Texto das mensagens anteriores oculto]

> [Texto das mensagens anteriores oculto]
> ----
> A Universidade Federal de São Paulo segue a política de e-mail Institucional disponível em https://sti.unifesp.br/documentos/termos-de-uso-do-email

--
Izadora de Souza

Mestranda em Hematologia e Oncologia - PPGHO

Escola Paulista de Medicina – EPM/UNIFESP

DREAM Lab (Drug REsistance And Mutagenesis Lab)

[Texto das mensagens anteriores oculto]

Matheus Molina <matheusmolina95@gmail.com> 23 de junho de 2022 13:31
Para: CLARISSA RIBEIRO REILY ROCHA <clarissa.rocha@unifesp.br>
Cc: izadora.souza@unifesp.br, Linda Seregni <linda.seregni@unifesp.br>, Camila Guedes <cguedes16@hotmail.com>, MARINA ANDRADE TOMAZ <marina.tomaz@unifesp.br>, Bruna Contieri <brunacontieri@hotmail.com>, Marcela Latancia <marcela.latancia@gmail.com>, Bruna Maldonado <brumaldonado@gmail.com>, Davi Mendes <davimendes4@gmail.com>, MARIANA LAZARINI <lazarini@unifesp.br>, Luciana Gomes <luciana.gomes@butantan.gov.br>

Dear Dr. Rocha,

I agree with both changes.

Best,
Matheus Molina Silva

[Texto das mensagens anteriores oculto]

> [Texto das mensagens anteriores oculto]
> [Texto das mensagens anteriores oculto]

Bruna Contieri <brunacontieri@hotmail.com> 23 de junho de 2022 13:32
Para: CLARISSA RIBEIRO REILY ROCHA <clarissa.rocha@unifesp.br>, "izadora.souza@unifesp.br" <izadora.souza@unifesp.br>, Linda Seregni <linda.seregni@unifesp.br>, Camila Guedes <cguedes16@hotmail.com>, Matheus Molina <matheusmolina95@gmail.com>, MARINA ANDRADE TOMAZ <marina.tomaz@unifesp.br>, Marcela

Latancia <marcela.latancia@gmail.com>, Bruna Maldonado <brumaldonado@gmail.com>, Davi Mendes <davimendes4@gmail.com>, MARIANA LAZARINI <lazarini@unifesp.br>, Luciana Gomes <luciana.gomes@butantan.gov.br>

Dear Clarissa,

I agree to both changes.

Thank you.

Best Regards,
Bruna Contieri

Obter o Outlook para Android

From: CLARISSA RIBEIRO REILY ROCHA <clarissa.rocha@unifesp.br>
Sent: Thursday, June 23, 2022 1:11:17 PM
To: izadora.souza@unifesp.br <izadora.souza@unifesp.br>; Linda Seregni <linda.seregni@unifesp.br>; Camila Guedes <cguedes16@hotmail.com>; Matheus Molina <matheusmolina95@gmail.com>; MARINA ANDRADE TOMAZ <marina.tomaz@unifesp.br>; Bruna Contieri <brunacontieri@hotmail.com>; Marcela Latancia <marcela.latancia@gmail.com>; Bruna Maldonado <brumaldonado@gmail.com>; Davi Mendes <davimendes4@gmail.com>; MARIANA LAZARINI <lazarini@unifesp.br>; Luciana Gomes <luciana.gomes@butantan.gov.br>
Subject: Fwd: CDDIS-21-5003RRR Initial Quality Check

[Texto das mensagens anteriores oculto]
[Texto das mensagens anteriores oculto]

Camila Guedes <cguedes16@hotmail.com> 23 de junho de 2022 13:35
Para: Bruna Contieri <brunacontieri@hotmail.com>, CLARISSA RIBEIRO REILY ROCHA <clarissa.rocha@unifesp.br>, "izadora.souza@unifesp.br" <izadora.souza@unifesp.br>, Linda Seregni <linda.seregni@unifesp.br>, Matheus Molina <matheusmolina95@gmail.com>, MARINA ANDRADE TOMAZ <marina.tomaz@unifesp.br>, Marcela Latancia <marcela.latancia@gmail.com>, Bruna Maldonado <brumaldonado@gmail.com>, Davi Mendes <davimendes4@gmail.com>, MARIANA LAZARINI <lazarini@unifesp.br>, Luciana Gomes <luciana.gomes@butantan.gov.br>

Dear Clarissa Rocha,

I agree to both changes.

Best Regards,

Camila Guedes

Obter o Outlook para Android

From: Bruna Contieri <brunacontieri@hotmail.com>
Sent: Thursday, June 23, 2022 1:32:48 PM
To: CLARISSA RIBEIRO REILY ROCHA <clarissa.rocha@unifesp.br>; izadora.souza@unifesp.br <izadora.souza@unifesp.br>; Linda Seregni <linda.seregni@unifesp.br>; Camila Guedes <cguedes16@hotmail.com>; Matheus Molina <matheusmolina95@gmail.com>; MARINA ANDRADE TOMAZ <marina.tomaz@unifesp.br>; Marcela Latancia <marcela.latancia@gmail.com>; Bruna Maldonado <brumaldonado@gmail.com>; Davi Mendes <davimendes4@gmail.com>; MARIANA LAZARINI <lazarini@unifesp.br>; Luciana Gomes <luciana.gomes@butantan.gov.br>
Subject: Re: CDDIS-21-5003RRR Initial Quality Check

[Texto das mensagens anteriores oculto]

Luciana Rodrigues Gomes <luciana.gomes@butantan.gov.br> 23 de junho de 2022 13:36
Para: Camila Guedes <cguedes16@hotmail.com>
Cc: Bruna Contieri <brunacontieri@hotmail.com>, CLARISSA RIBEIRO REILY ROCHA <clarissa.rocha@unifesp.br>, "izadora.souza@unifesp.br" <izadora.souza@unifesp.br>, Linda Seregni <linda.seregni@unifesp.br>, Matheus Molina <matheusmolina95@gmail.com>, MARINA ANDRADE TOMAZ <marina.tomaz@unifesp.br>, Marcela Latancia <marcela.latancia@gmail.com>, Bruna Maldonado <brumaldonado@gmail.com>, Davi Mendes <davimendes4@gmail.com>, MARIANA LAZARINI <lazarini@unifesp.br>

Dear Clarissa,

I agree with both changes.

Thank you.

Best regards,

Luciana R Gomes
[Texto das mensagens anteriores oculto]
--
Luciana Rodrigues Gomes, Ph.D
Research Associate
Center for Research on Toxins, Immune-Response and Cell Signaling (CeTICS)
Laboratório Especial de Ciclo Celular - LECC
Av. Dr Vital Brasil, 1500, Prédio 105 - Butantã
CEP: 05503-001, Instituto Butantan, São Paulo, SP, Brazil
email: luciana.gomes@butantan.gov.br

Esta mensagem, incluindo seus anexos, tem caráter confidencial e seu conteúdo é restrito ao destinatário da mensagem. Se você não for o(a) destinatário(a) final ou recebeu esta mensagem por engano, você não pode copiar, utilizar, divulgar ou agir baseado nesta mensagem ou qualquer informação nela contida. Por gentileza comunique imediatamente o remetente desta mensagem e a remova de seu sistema. Qualquer uso não autorizado, replicação ou disseminação desta mensagem ou parte dela é expressamente proibido. As informações ou opiniões pessoais do remetente nela contida podem não refletir o ponto de vista do Instituto / Fundação que apenas é divulgado por colaboradores devidamente autorizados. Eventuais Dados Pessoais contidos nesta mensagem, estão protegidos e em conformidade com a Lei Geral de Proteção de Dados - LGPD.

The contents of this email message and any attachments are intended solely for the addressee(s) and may contain confidential information. If you are not the intended recipient of this message, or if this message has been addressed to you in error, you may not copy, use, disseminate nor act based on this message or any information contained herein. Please immediately alert the sender by reply email and then delete this message and any attachments from the system. You are hereby notified that any unauthorized use, copying or dissemination of this message, in whole or in part, is strictly prohibited. The information or personal opinions of the sender contained herein may not reflect the point of view of the Institute/Foundation, which is only disclosed by duly authorized associates. Any Personal Data contained in this message is protected and in compliance with the Brazilian General Data Protection Law (LGPD).

MARIANA LAZARINI <lazarini@unifesp.br> 23 de junho de 2022 13:45
Para: Luciana Rodrigues Gomes <luciana.gomes@butantan.gov.br>
Cc: Camila Guedes <cguedes16@hotmail.com>, Bruna Contieri <brunacontieri@hotmail.com>, CLARISSA RIBEIRO REILY ROCHA <clarissa.rocha@unifesp.br>, Izadora Souza <izadora.souza@unifesp.br>, Linda Seregni <linda.seregni@unifesp.br>, Matheus Molina <matheusmolina95@gmail.com>, MARINA ANDRADE TOMAZ <marina.tomaz@unifesp.br>, Marcela Latancia <marcela.latancia@gmail.com>, Bruna Maldonado <brumaldonado@gmail.com>, Davi Mendes <davimendes4@gmail.com>

Dear Dr. Rocha,

I agree with both changes.

Best,

Mariana Lazarini
[Texto das mensagens anteriores oculto]

Bruna Maldonado <brumaldonado@gmail.com> 23 de junho de 2022 13:56
Para: CLARISSA RIBEIRO REILY ROCHA <clarissa.rocha@unifesp.br>
Cc: izadora.souza@unifesp.br, Linda Seregni <linda.seregni@unifesp.br>, Camila Guedes <cguedes16@hotmail.com>, Matheus Molina <matheusmolina95@gmail.com>, MARINA ANDRADE TOMAZ <marina.tomaz@unifesp.br>, Bruna Contieri <brunacontieri@hotmail.com>, Marcela Latancia <marcela.latancia@gmail.com>, Davi Mendes <davimendes4@gmail.com>, MARIANA LAZARINI <lazarini@unifesp.br>, Luciana Gomes <luciana.gomes@butantan.gov.br>

Dear Clarissa and collegues,

I agree with both changes!

Best,

Bruna

[Texto das mensagens anteriores oculto]
[Texto das mensagens anteriores oculto]
[Texto das mensagens anteriores oculto]

Davi Mendes <davimendes4@gmail.com> 23 de junho de 2022 15:29
Para: Bruna Maldonado <brumaldonado@gmail.com>
Cc: CLARISSA RIBEIRO REILY ROCHA <clarissa.rocha@unifesp.br>, Izadora de Souza <izadora.souza@unifesp.br>, Linda Seregni <linda.seregni@unifesp.br>, Camila Guedes <cguedes16@hotmail.com>, Matheus Molina <matheusmolina95@gmail.com>, MARINA ANDRADE TOMAZ <marina.tomaz@unifesp.br>, Bruna Contieri <brunacontieri@hotmail.com>, Marcela Latancia <marcela.latancia@gmail.com>, MARIANA LAZARINI <lazarini@unifesp.br>, Luciana Gomes <luciana.gomes@butantan.gov.br>

Dear Clarissa and collegues,

I agree with both changes!

Best,

DM
[Texto das mensagens anteriores oculto]

Linda Seregni <linda.seregni@unifesp.br> 23 de junho de 2022 15:34
Para: Davi Mendes <davimendes4@gmail.com>
Cc: Bruna Maldonado <brumaldonado@gmail.com>, CLARISSA RIBEIRO REILY ROCHA <clarissa.rocha@unifesp.br>, Izadora de Souza <izadora.souza@unifesp.br>, Camila Guedes <cguedes16@hotmail.com>, Matheus Molina <matheusmolina95@gmail.com>, MARINA ANDRADE TOMAZ <marina.tomaz@unifesp.br>, Bruna Contieri <brunacontieri@hotmail.com>, Marcela Latancia <marcela.latancia@gmail.com>, MARIANA LAZARINI <lazarini@unifesp.br>, Luciana Gomes <luciana.gomes@butantan.gov.br>

Dear Clarissa and collegues,

I agree with both changes!

Best,

Linda

[Texto das mensagens anteriores oculto]

Marina Andrade Tomaz <marina.tomaz@unifesp.br> 24 de junho de 2022 10:11
Para: Linda Seregni <linda.seregni@unifesp.br>
Cc: Davi Mendes <davimendes4@gmail.com>, Bruna Maldonado <brumaldonado@gmail.com>, CLARISSA RIBEIRO REILY ROCHA <clarissa.rocha@unifesp.br>, Izadora de Souza <izadora.souza@unifesp.br>, Camila Guedes <cguedes16@hotmail.com>, Matheus Molina <matheusmolina95@gmail.com>, Bruna Contieri <brunacontieri@hotmail.com>, Marcela Latancia <marcela.latancia@gmail.com>, MARIANA LAZARINI <lazarini@unifesp.br>, Luciana Gomes <luciana.gomes@butantan.gov.br>

Dear Clarissa and collegues,

I agree with both changes!

Best,

[Texto das mensagens anteriores oculto]
